# Insights into microbial compositions of the respiratory tract of neonatal dairy calves in a longitudinal probiotic trial through 16S rRNA sequencing

**DOI:** 10.3389/fmicb.2024.1499531

**Published:** 2025-01-08

**Authors:** Jia W. Tan, Susan D. Eicher, Janice E. Kritchevsky, Keith A. Bryan, Aaron Dickey, Carol G. Chitko-McKown, Tara G. McDaneld

**Affiliations:** ^1^USDA, ARS, U.S. Meat Animal Research Center, Clay Center, NE, United States; ^2^Livestock Behavior Research Unit, USDA, ARS, West Lafayette, IN, United States; ^3^Department of Veterinary Clinical Sciences, College of Veterinary Medicine, Purdue University, West Lafayette, IN, United States; ^4^Chr. Hansen, Inc., Milwaukee, WI, United States

**Keywords:** dairy cattle, 16S rRNA, microbiome, probiotic, respiratory

## Abstract

**Introduction:**

Probiotics are a promising intervention for modulating the microbiome and the immune system, promoting health benefits in cattle. While studies have characterized the calf lung bacterial profile with and without oral probiotics, simultaneous probiotic effects on the bacterial populations of multiple sites along the respiratory tract have not been characterized.

**Methods:**

This study utilized the same pre-weaning diary calf group from our previous studies to characterize the bacterial populations present in the nostril and tonsil across control and treatment groups and nine sampling time points. DNA was exacted from the nostril and tonsil swabs and lung lavage fluids, and 16S ribosomal RNA gene hypervariable regions 1-3 were subsequently sequenced.

**Results:**

Temporal variation in alpha bacterial diversity within the nostril, tonsil, and lung samples was observed, indicating distinct bacterial compositions among sampling time points. Oral probiotic treatment did not change alpha diversity in any respiratory tissue, however, spatial variability in bacterial taxa composition was observed among the three respiratory tract regions. While the majority of differentially abundant taxa in probiotic treated calves were unique to their anatomical location, a few were common to two anatomical locations and one *Finegoldia* amplicon sequence variant was differentially abundant in all three anatomical locations.

**Discussion:**

In conclusion, these findings contribute to the understanding of the dynamic nature of bacterial diversity and the potential effects of probiotics within the bovine respiratory tract and provides insight for future studies of probiotics on animal health, disease prevention, and management.

## Introduction

Cattle are subject to various health challenges that can impact both animal welfare and economic sustainability in the livestock industry. Among the numerous factors influencing cattle health, the microbiome—the diverse community of microorganisms inhabiting various anatomical sites—plays a crucial role in maintaining host health, immune function, and metabolic processes (Al-Shawi et al., 2020). Feed additives such as probiotics have emerged as promising interventions for modulating the microbiome and promoting health benefits in cattle and other livestock species (Alayande et al., 2020). The beneficial effects of probiotics have been reported on both physiological and molecular levels in cattle, such as increased feed intake and daily weight gain, influencing gut epithelial gene expression, promoting barrier function, and modulating inflammatory responses (Alugongo et al., 2017; Petri et al., 2018; Huebner et al., 2019; Ban and Guan, 2021). Direct-fed microbials have also been shown to improve health outcomes (McAllister et al., 2011) and immune responses (Buntyn et al., 2016; McAllister et al., 2011). Moreover, the beneficial effects of several bacterial species, such as *Lactobacillus*, *Enterococcus*, and *Bifidobacterium* have been reported (Adjei-Fremah et al., 2018; Mountzouris et al., 2007). While the exact mechanisms of action of probiotics are not well elucidated, it was proposed that their involvement in gastrointestinal modification played a crucial role (Satokari, 2019). Therefore, it was not surprising to see the optimization effect in ruminal fermentation, enhancement in fiber degradation, and improvement in the efficiency of microbial protein synthesis in cattle with the proper probiotic usage (DeVries and Chevaux, 2014; Yuan et al., 2015; Dias et al., 2018).

The effects of oral probiotics have been extensively studied for their impact on ruminal fermentation, gastrointestinal microbiota, intestinal tract function and integrity, and feces; however, limited research has explored their effects on the microbiome of the bovine respiratory tract. Still, some studies have reported the impact of probiotics on respiratory disease in livestock species. In a calf respiratory challenge study where calves were fed an effective oral probiotic prior to an infectious bovine rhinotracheitis intra-nasal challenge followed by a *Mannheimia haemolytica* intra-nasal challenge, more probiotic-fed calves survived the challenge compared with controls (Bryan et al., unpublished data). Moreover, a few studies have found beneficial outcomes of feeding effective probiotics on responses of sows and piglets to Porcine Reproductive and Respiratory Syndrome (Lerner et al., 2020; Bertram et al., 2022).

The complexity of the respiratory tract microbiome and its impact on disease prevention cannot be understated; unfortunately, it is poorly understood currently. Lungs were previously believed to be a sterile environment, however, recent studies have shown contrasting results, where the respiratory microbiota plays a role in regulating the activation of both the innate and adaptive immune responses (Dickson et al., 2016). Moreover, research has shown that a healthy microbiome in one organ system, namely the gut, can affect the health of other systems through the common mucosal immune system (Welch et al., 2022). An example of such crosstalk is between the gut and respiratory microbiota, known as the gut-lung axis, has been reported to influence the immune function and inflammatory responses in various species (e.g., Chase, 2018; Saint-Martin et al., 2022).

The animal population used in this study was previously described by Eicher et al. (2023) and McDaneld et al. (2024) where probiotic treatment altered immune cell populations and the lung bacterial population composition, respectively. This study aimed to expand on the characterization of the bacterial populations present in the nostril, tonsil, and lung regions of the respiratory tract; thus, providing further insights into how probiotics influence the respiratory microbiome, and their potential role in promoting respiratory health and immunity in cattle.

## Materials and methods

### Animal populations and sample collection

As described previously in Eicher et al. (2023) and McDaneld et al. (2024), data collection occurred in 2018 at the Purdue University Dairy Teaching and Research Unit, with approval from the Purdue Animal Care and Use Committee (#1803001701). Twenty Holstein calves, meeting criteria of birth weight between 32 and 50 kg and plasma protein value ≥5.5 g/dL, measured by Brix refractometry within 24–48 h post-birth, were included. Calves received 1 L of colostrum within 12 h of birth and again within 24 h, followed by 2 L of 24/20 milk replacer (Milk Specialties Global, Eden Prairie, MN, USA), divided into two equal feedings per day. From day 2 of life, calves that were randomly assigned to treatments at birth were moved to individual hutches and assigned to probiotics (Treatment, *n* = 10) or control (Control, *n* = 10) groups. Probiotics (Bovamine Dairy, Chr. Hansen, Inc., Milwaukee, WI, USA) were added to each bottle (2.5 g/bottle, two bottles/day), and they were kept refrigerated until use. The probiotics contained lactose, sodium silico aluminate, and live (viable), naturally occurring microorganisms including dried *Propionibacterium freudenreichii* fermentation product, and dried *Lactobacillus animalis* fermentation product (guaranteed at 1.5 × 10^9^ CFU/g). Calves were weaned (step-down) gradually at day 42; meaning one milk feeding was discontinued at day 42 and the second discontinued based on dry feed consumption (approximately 1.5 kg/day). Probiotics were added to the dry feed at a targeted intake of approximately 5 g/day from day 7 until after weaning was completed. Dry feed was available from day 7 to 52. Individual calf weights were recorded weekly until day 49, with weigh days assigned based on their birth date. Additionally, calves were monitored daily for fecal scores (Eicher-Pruiett et al., 1992), ocular and nostril discharge, ear orientation and overall clinical score. None of the calves in the study were diagnosed or treated for respiratory disease during the duration of the study.

Nostril and tonsil swabs were obtained from the upper respiratory tract of all calves on day 0, 7, 14, 21, 28, 35, 42 and 49 of the study. These samples will be referred to as nostril and tonsil throughout the manuscript. Prior to nostril sampling, any fecal material present on the animal’s nose was wiped clean using a single-use towel. A single unguarded 15.24 cm nostril swab was gently inserted into each nostril cavity to a depth of approximately 14 cm, rotated, and then removed. Following sample collection, the swabs were placed in buffered peptone water with 12% glycerol for subsequent bacterial taxa evaluation. For tonsil sampling, the calves’ mouths were held open, and their tongues were positioned to the side by hand. A swab was inserted and moved back and forth against the left tonsil, followed by the right tonsil, and then removed from the calves’ mouths. These swabs were also placed in buffered peptone water with 12% glycerol, snap frozen in liquid nitrogen immediately after collection, and stored at −80°C until DNA extraction and sequence library preparation for subsequent bacterial taxa assessment. Buffered peptone water with 12% glycerol was originally selected as a storage media as there was interest in the option of plating samples on selective media plates. Upon collection of the samples and initial analysis of the sequence data, there was decreased interest in plating the samples and our focus changed to the 16S rRNA gene sequence data.

On day 52 of the study, bronchoalveolar lavages were conducted on five calves randomly selected from the 10 calves in each treatment group. These samples will be referred to as lung throughout the manuscript. The smaller lung sample size was due to the invasiveness of the technique used to collect the samples. Prior to the procedure, cetacaine (Cetylite Inc., Pennsauken, NJ, USA) was sprayed into the left nostril of the calf after relocating them to the barn near their housing area. While calves were gently restrained by two people, the end of a bovine bronchoscope (Olympus OSF-2 Flexible sigmoidoscope) was sprayed with cetacaine and inserted through the nostril and into the trachea. A flexible 10 French catheter (36″ in length) was inserted through the bronchoscope and 120 mL of sterile saline at 37°C was infused into the lungs using 60-mL syringes. Immediately after the 120-mL infusion, negative pressure was applied to aspirate the fluid back through the catheter and into a sterile 50-mL endotoxin free centrifuge tube. The process was repeated to obtain a second sample if necessary to obtain a total of 50 mL of lavage fluid. Samples were placed on ice and then stored at −80°C. These samples detailed above were used for evaluation of the bacterial microbiome through amplification and sequencing of the 16S rRNA gene.

### DNA extraction and 16S rRNA gene amplification library preparation and sequencing

Total DNA was extracted from all samples using a commercial kit (PowerSoil DNA kit; Qiagen, Germantown, MD, USA) following the standard protocol and initial DNA quantity was evaluated with a DNA spectrophotometer (DeNovix DS-11 FX Series; Wilmington, DE, USA). PCR-grade water was used as the negative control and processed with the other samples in the DNA extraction process to evaluate contamination in the kit reagents. Amplicon library preparation was performed by amplification of the 16S rRNA gene V1-V3 hypervariable region for each DNA sample using standard PCR (AccuPrime, Invitrogen, Carlsbad, CA, USA) and primers with index sequences as previously described that amplify hypervariable regions 1 through 3 of the 16S rRNA gene (Myer et al., 2015). Quality and quantity of the resulting 16S rRNA gene amplification was checked on the Fragment Analyzer (Advanced Analytical, Ankeny, IA, USA). By using indexed primers to amplify the 16S rRNA gene, individual samples were pooled and then sequenced utilizing the MiSeq Illumina Sequencer (Illumina, San Diego, CA, USA) with a MiSeq Reagent Kit v3 to generate 2 × 300 paired end reads at the US Meat Animal Research Center Core Lab. Samples (*n* = 10) that did not pass the initial quality score cutoff of Q20 > 75% for sequence reads were run in a second sequencing run and the data were combined across sequencing runs.

### Data analysis

#### Qiime2 workflow

Sequencing data obtained from the Illumina MiSeq sequencer were initially processed using the Quantitative Insights Into Microbial Ecology 2 (Qiime2; Bolyen et al., 2019) pipeline (version 2023.3.2 at the time of data processing; Supplementary File 1). Raw sequencing reads and re-sequenced samples were quality-filtered, trimmed, denoised, and chimeras were filtered out using the DADA2 plugin (Callahan et al., 2016) separately, and the resulting feature table representative sequences were merged after (feature-table merge and feature-table merge-seqs options, respectively), which resulted in a combined dataset of amplicon sequence variants (ASVs). Taxonomic classification of the merged sequences was performed at all taxonomic levels using a pre-trained classifier with the Greengenes2 2022.10 dataset^1^ (McDonald et al., 2023). The Greengenes2 database was selected as it was the most recently updated database at the time the data herein were analyzed. Greengenes2 is larger than past resources in its phylogenetic coverage, as compared to SILVA, Greengenes and Genome Taxonomy Database (GTDB; McDonald et al., 2023). Shannon Diversity Index was used to estimate ASV diversity, which accounts for both abundance and evenness of the taxa present (Shannon, 1949). Additionally, Qiime2 default outputs also include measures of: Faith’s phylogenetic Diversity measure to estimate ASV diversity (Faith, 1992); Observed Features to qualitatively measure community richness; and Pielou’s evenness to measure community evenness (Pielou, 1966). Unweighted Unifrac distance was used to qualitatively measure the community dissimilarity incorporating phylogenetic relationships between the features (Lozupone and Knight, 2005) coupled with standard multivariate statistical analyses such as principal coordinates analysis (PCoA) and distance based permutational analysis of variance (PERMANOVA). Moreover, Qiime2’s default outputs for beta diversity measures included Jaccard (Jaccard, 1912), Bray-Curtis (in the adonis() function and vegan package; Sorensen, 1948) and weighted Unifrac (Lozupone et al., 2007) distances. The resulting Qiime2 artifacts such as metadata, feature table, constructed phylogenetic tree using MAFFT fasttree option (Katoh et al., 2002; Price et al., 2010), alpha and beta diversity measures, and ASV table were exported for downstream visualization and analysis.

#### R workflow

The exported Qiime2 artifacts generated from the Qiime2 pipeline were imported into R environment (Version 4.2.1; R Core Team, 2019; Supplementary File 2) and visualized using the R package qiime2R (Bisanz, 2018) following the standard protocols. Differential abundance was evaluated using the package ANCOM-BC2 (Lin and Peddada, 2020, 2024) with adapted parameters including 10% prevalence inclusion criteria, 95% confidence level, structural zero detection, regularization factor of 5%, and bootstrap level of 100. A random intercept was introduced that accounts for the random effect in ANCOM-BC2. The Holm-Bonferroni method (p_adj_method = “holm”) was used as the default to adjust *p*-values was used as the default to adjust *p*-values and corrects for the familywise error rate (Holm, 1979). Moreover, treatment (control or treatment) was used as the “group” variable for downstream pairwise comparisons. Differential abundance analysis results were visualized in bar plot format to visualize the log fold-changes with the R package ggplot2 (Wickham, 2011). Results obtained from both Qiime2 and R workflows were interpreted together to gain comprehensive insights into the microbial community structure and composition across different sampling locations (nostril, tonsil, lung) in calves. Visualization such as heatmaps, relative abundance plots, line graphs, and PCoA plots with ggplot2 were employed to illustrate the relationships between samples and identify key microbial taxa associated with specific anatomical locations.

## Results

### Summary of output sequence

For the sequenced libraries, a total of 66,399,042 reads were generated (Supplementary Table 1). An evaluation of the individual libraries resulted in a minimum of 26,629, a maximum of 1,042,424, and an average of 220,594.8 reads (Supplementary Table 1). Proportion of reads classified at each taxonomic level are presented at the phylum, class, order, family, and genus level (Supplementary Table 2). After processing of the reads, 136,551 ASV were inferred from the dataset and classified into 1,987 unique genera.

### Alpha diversity

**Figure 1:**
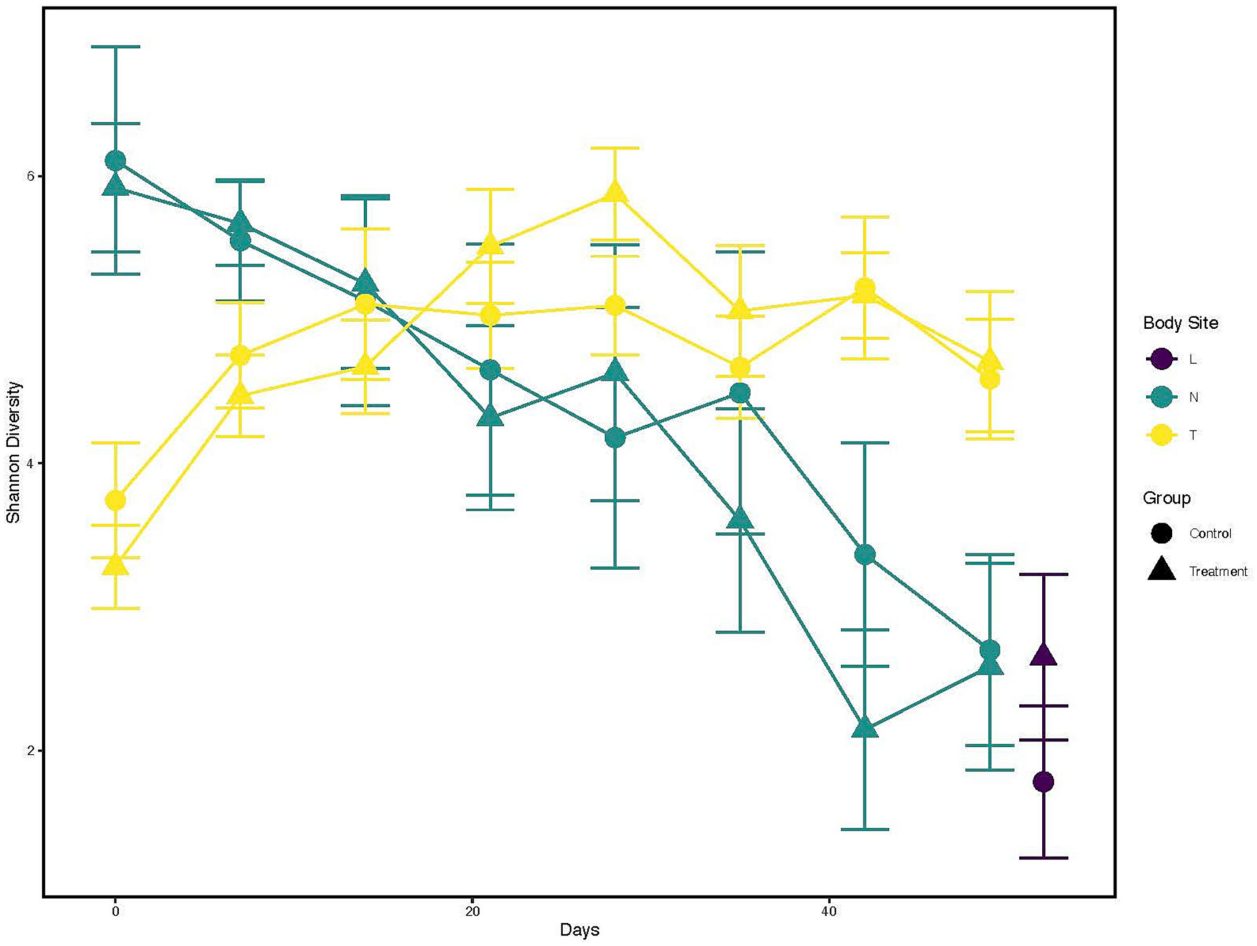
Alpha diversity changes over time and across different anatomical locations, and probiotic treatment groups. X-axis indicates the different time points a sample was collected. While the Y-axis represents Shannon Index value. The color of the line represents the anatomical locations; Yellow: Tonsil samples, Green: Nostril samples, Purple: Lung samples. The shapes represents whether a probiotic was fed; Dot: Control samples, Triangle: Probiotic treatment.

The Shannon diversity index was calculated and visualized to represent the temporal change across sampling timepoints and difference in diversity between treatment groups within each anatomical location (Figure 1; Supplementary Table 3). Nostril samples showed similar and high (Shannon index >5) diversity at days 0, 7 and 14 for both control and treated calves. Diversity began to decrease for both calf groups post day 14 (Shannon index <4 by day 49).

Contrary to nostril samples, a general upward trend in bacterial taxa diversity for tonsil samples was observed (Figure 1, yellow line; Supplementary Table 3) for both control and treated calves. The diversity from tonsil samples at day 0 was lower than from nostril samples (Shannon index <4). The diversity index increased post day 0 for both control and treated tonsil samples (Shannon index ~5 by day 28). Tonsil samples from control calves showed a trend to higher diversity than from treated calves at day 0, 7, and 14. Tonsil samples from treated calves then began to show higher diversity trend compared to control calves between days 21 and 35. Lung samples trended toward a higher diversity from treated calves (Shannon index >2) than from control calves (Shannon index <2).

Finally, a Kruskal-Wallis test followed by a pairwise comparison of the Shannon index across anatomical locations showed a significant difference in microbial diversity. Comparisons of nostril and lung (adjusted *p* < 0.005) and tonsil and lung (adjusted *p* < 0.000006) showed a greater microbial diversity in nostril and tonsil for both comparisons to lung, but a similar microbial diversity for the nostril and tonsil comparison (adjusted *p* = 0.20; Supplementary Figure 1). Overall alpha diversity between all control and treated samples at each anatomical location did not show statistical significance (*p* = 0.84; data not shown).

### Beta diversity

**Figure 2:**
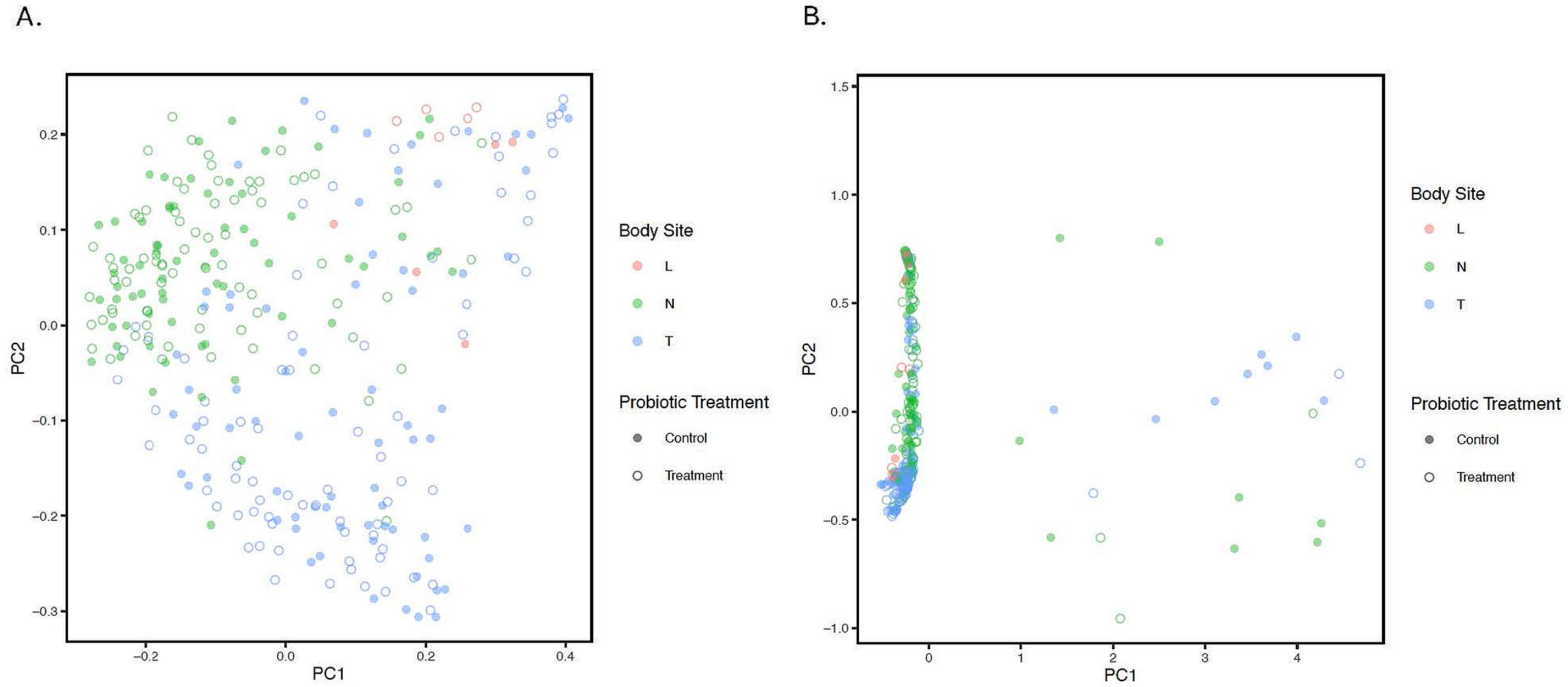
Principle Coordinate Analysis (PCoA) of unweighted **(A)** and weighted **(B)**. UniFrac distances illustrating variation in microbial community structure associated with anatomical location and probiotic treatment groups. The PCoA demonstrates the clustering of 16S rRNA gene sequences from samples collected at different anatomical locations and by treatment. Different colors represent the location; Blue: Tonsil samples, Green: Nostril samples, Red: Lung samples. While solid circles represent the control samples, open unfilled circles represent treated samples from treated calves. Statistical difference was observed for lung-tonsil, tonsil-nostril, and nostril-lung samples (adjusted *p* < 0.001).

The PCoA plot was constructed based on the unweighted and weighted UniFrac distance to represent variation in bacterial profiles across nostril, tonsil, and lung samples (beta diversity). The PERMANOVA/adonis test on the distance values indicated a significant difference for each among anatomical site comparison while stratifying for repeated measures (‘Animal’) (all adjusted *p* < 0.001, *R*^2^ value = 0.07; Figure 2; Supplementary Figure 2). Moreover, statistical significance was observed for samples from all treated calves compared to samples from all control calves regardless of the anatomical groups (adjusted *p* = 0.041; Supplementary Figure 3). PERMDISP was performed to confirm the significant results observed in the unweighted UniFrac distance is not caused by a difference in dispersion (*p* > 0.05).

**Figure 3:**
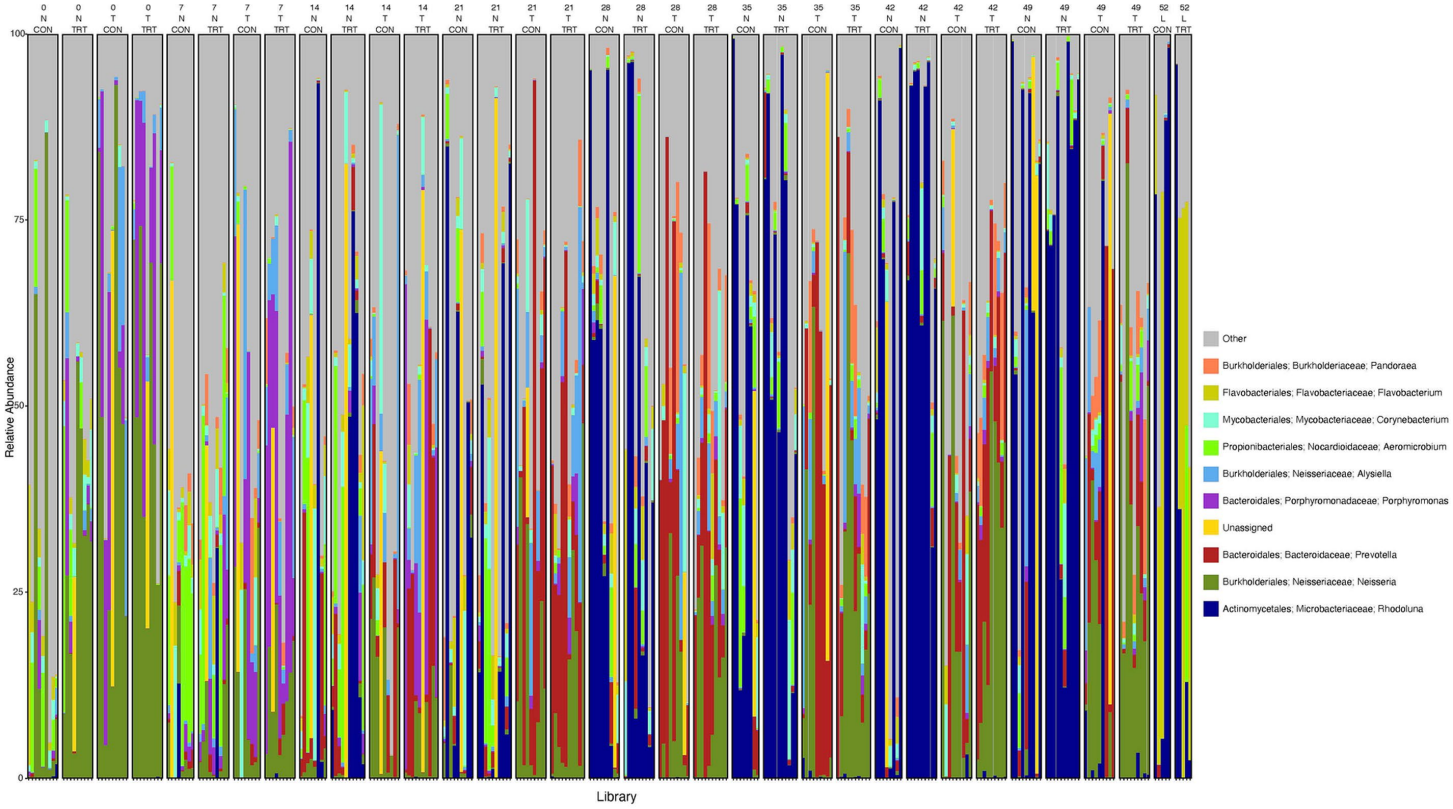
Relative abundance profile of top 10 microbial taxa at the genus level separated by sampling time point, anatomical locations, and probiotic treatment. The top 10 most abundant taxa with the relative abundance of ≥1% have been selected to show the distribution change over time (day 0, 7, 14, 21, 28, 35, 42 and 49 for nostril and tonsil and day 52 for lung), in different anatomical locations (N = nostril, T = tonsil, and L = lung), and on probiotic treatment (CON = control and TRT = treatment). Taxa that could not be classified to the genus level are grouped as unclassified with the remaining genera of low abundance identified as other. Classification in the legend is from order to genus.

**Figure 4:**
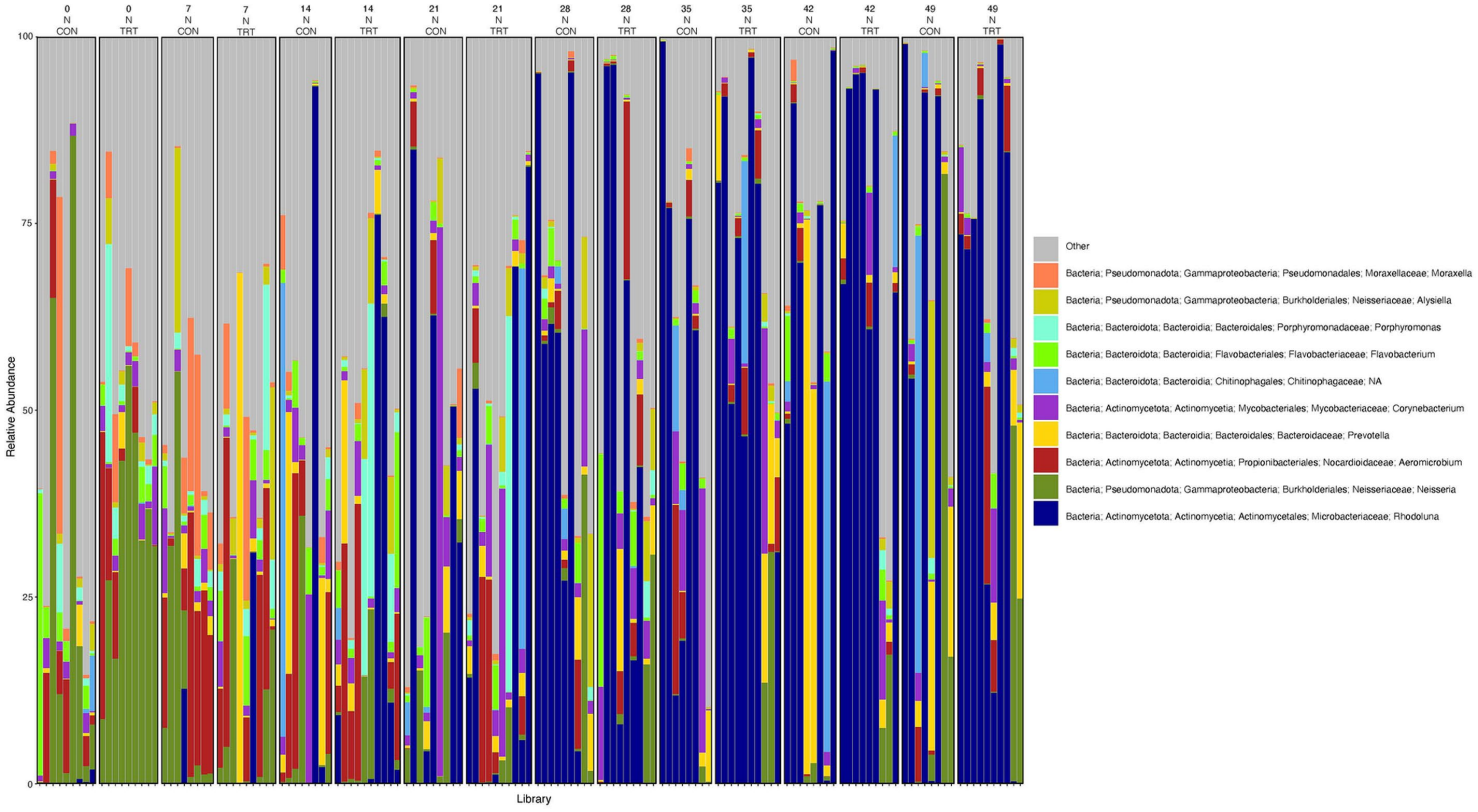
Relative abundance profile of top 10 microbial taxa at the genus level for nostril separated by sampling time point and probiotic treatment. The top 10 most abundant taxa with the relative abundance of ≥1% have been selected to show the distribution change over time (day 0, 7, 14, 21, 28, 35, 42 and 49) and probiotic treatment (CON = control and TRT = treatment) at the nostril sampling site (N = nostril). Remaining genera of low abundance identified as other. Classification in the legend is from kingdom to genus.

**Figure 5:**
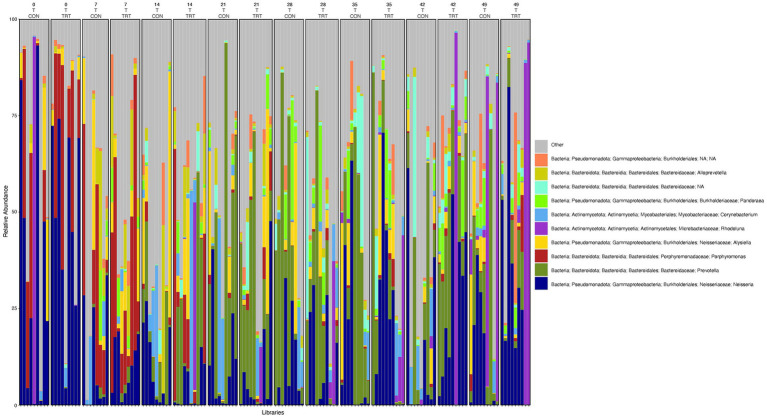
Relative abundance profile of top 10 microbial taxa at the genus level for tonsil separated by sampling time point and probiotic treatment. The top 10 most abundant taxa with the relative abundance of ≥1% have been selected to show the distribution change over time (day 0, 7, 14, 21, 28, 35, 42 and 49) and probiotic treatment (CON = control and TRT = treatment) at the tonsil sampling site (T = tonsil). Remaining genera of low abundance identified as other. Classification in the legend is from kingdom to genus.

**Figure 6:**
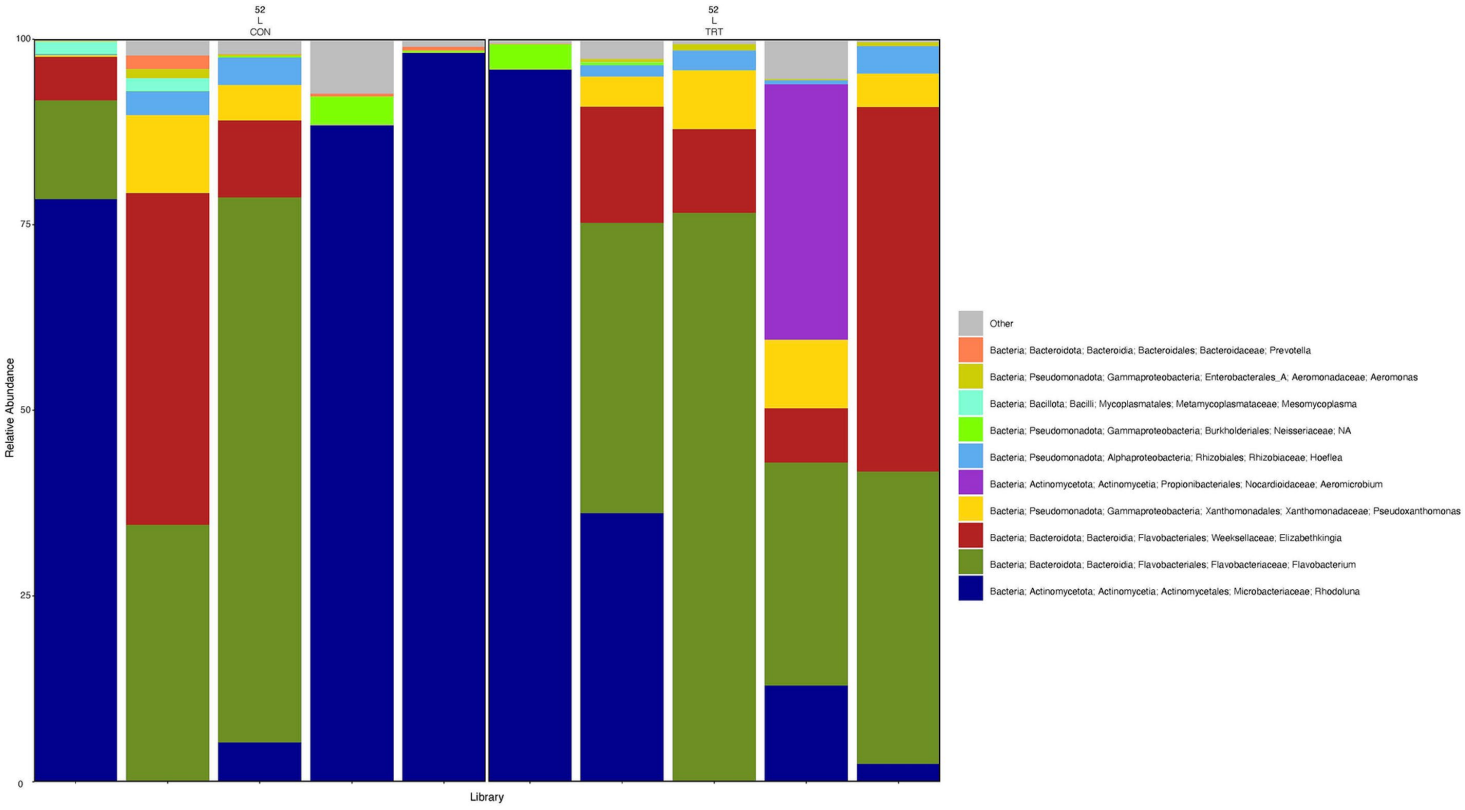
Relative abundance profile of top 10 microbial taxa at the genus level for lung separated by probiotic treatment. The top 10 most abundant taxa with the relative abundance of ≥1% have been selected to show the distribution change over probiotic treatment (CON = control and TRT = treatment) at the lung sampling site (L = lung). Remaining genera of low abundance identified as other. Classification in the legend is from kingdom to genus.

To gain a general idea of the overall abundance pattern of multiple bacterial taxa across multiple groups of samples, a heatmap of ASVs with >1% relative abundance was constructed (Supplementary Figure 4) based on sample time, anatomical locations, and treatment groups. Subsequently, the top 10 ASVs with >1% relative abundance were selected to further explore the effect of temporal changes, anatomical locations, and treatments on the distribution of those specific bacterial taxa within and between sample types. A relative abundance plot was generated to represent all time points and locations, separated by treatment groups (Figure 3). Relative abundance plots were also generated separately for nostril, tonsil, and lung sampling sites at all time points and separated by treatment groups (Figures 4–6, respectively). Average relative abundance of the ASVs for all sequence libraries within sampling site and timepoint are presented in Supplementary Table 4. The average relative abundance is represented as a percentage of 100. The temporal effect on the distribution of bacterial taxa is well represented in the plot, as genus *Neisseria* was predominately abundant in both treated and control nostril and tonsil samples at day 0, and the abundance diminishes over time. The spatial difference is demonstrated as well. For example, genus *Prevotella* is mostly abundant in tonsil samples, regardless of the sample time compared to nostril samples, whereas the order *Actinomycetales* is mostly abundant in nostril samples regardless of the sample time compared to tonsil samples. Similarly, *Flavobacterium* is mostly abundant in lung, and less present in nostril and tonsil samples, regardless of the sample time and treatment group. In addition, the bacterial genus *Aeromicrobium* was observed to be consistently abundant regardless of the sample time, anatomical locations, and the treatment groups. While the genus *Alysiella* appeared to be abundant in most samples, except for treated nostril samples on day 49 and treated lung samples on day 52.

### Differential abundance analysis

Differential abundance analysis was performed on all samples based on their corresponding timepoints and anatomical locations to elucidate the effect of feeding probiotic (Supplementary Tables 5–7; Supplementary Figures 5–7). Various taxa were found to be differentially abundant in both tonsil and nostril samples of control calves and treated calves (adjusted *p* < 0.05). For example, *Neisseria* was found to be differentially abundant in nostril on days 21 and 49 (decreased, treated versus controls), *Peptostreptococcus* on days 7 and 14 (increased, treated versus controls), and unclassified *Rhondothermales* on days 21, 42, and 49 (decreased, treated versus controls). For tonsil samples, unclassified *Pseudomonadaceae* was differentially abundant on days 7 and 42 (decreased, treated versus controls), family *Burkholderiaceae* on days 28 and 49 (increased and decreased, respectively, treated versus controls), unclassified *Sphingobacteriaceae* on days 7 (decreased, treated versus controls), 21 (increased, treated versus controls), and 35 (increased, treated versus controls), unclassified *Enterobacteriaceae* on days 7 and 42 (decreased, treated versus controls), *Neorhizobium* on days 35 and 42 (decreased, treated versus controls), *Falsochrobactrum* on days 7, 42, and 49 (decreased, treated versus controls), and *Andreesenia* on days 28 and 49 (increased, treated versus controls). Very few overlaps were observed for differential abundant taxa in nostril and tonsil samples (Supplementary Tables 5, 6; Supplementary Figures 5, 6). For example, *Neofamilia* was found to be differentially abundant in both tonsil and nostril samples on day 7 (increased, treated versus controls). Other taxa were differentially abundant in both sampling sites but were identified to be differentially abundant at different sampling times. *Kaistia* was identified to be differentially abundant on day 0 for nostril samples and day 14 for tonsil samples (decreased, treated versus controls), while *Phenylobacterium* was identified to be differentially abundant on day 7 for nostril samples and day 42 for tonsil samples (increased, treated versus controls). Some notable differentially abundant taxa identified in lung samples included *Moraxella* (increased, treated versus controls)*, Pseudomonas* (increased, treated versus controls)*, Fusobacterium* (decreased, treated versus controls)*, Acinetobacter* (decreased, treated versus controls)*, Pasteurella* (decreased, treated versus controls), and *Bacteroides* (decreased, treated versus controls) (Supplementary Table 7; Supplementary Figure 7). The genera *Finegoldia* was the only taxa found to be commonly differentially abundant in all three sampling niches: day 14 for nostril, day 21 for tonsil and day 52 for lung samples (increased, treated versus controls).

## Discussion

This study utilized 16S rRNA amplicon sequencing to characterize the bacterial community structure in different anatomical locations of the respiratory tract in pre-weaned dairy calves during the feeding of probiotics. The evaluation of alpha diversity of the bacterial profiles of different anatomical sites and sampling times, identified a clear temporal and partial spatial effect on alpha diversity in both nostril and tonsil samples, indicated by the variation of richness (number of taxa) and evenness as reported (the relative abundance of those taxa) over time within these anatomical sites. The significance of observed variation was confirmed by statistical analysis in richness and/or evenness between nostril-lung and tonsil-lung sample pairs, therefore suggesting distinct microbial composition between the upper respiratory tract (nostril and tonsil) and the lower respiratory tract (lung samples). Previous studies suggest that anatomical niches within the respiratory tract are likely to have different local environments (Chai et al., 2022; Zhang et al., 2023), therefore explaining the difference in microbial composition and structure observed in the upper and lower respiratory tract.

Despite temporal variation and difference between upper and lower respiratory tract samples, the overall alpha diversity between all control and treated samples at each anatomical location did not show statistical significance. This suggests that while there may be temporal fluctuations in bacterial diversity within specific anatomical sites (partial spatial effect), the overall richness and evenness of the bacterial taxa remain consistent regardless of probiotic treatment. Moreover, although lower alpha diversity has been reported to be associated with disease (e.g., Centeno-Martinez et al., 2022; Timsit et al., 2018; Holman et al., 2015) in the upper respiratory tract microbiome, the low diversity of the lung compared to other respiratory tract locations of post-weaned beef calves and feedlot cattle have not been associated with disease. Previous research has also reported the beneficial effects of probiotics in the gut of cattle with the improvement of feed intake, weight gain, efficiency of microbial protein synthesis and immune response (DeVries and Chevaux, 2014; Yuan et al., 2015; Alugongo et al., 2017; Dias et al., 2018; Petri et al., 2018; Huebner et al., 2019; Ban and Guan, 2021). As for the role of probiotics in the respiratory tract of cattle, Alayande et al. (2020) reported that probiotics impact the microbiome and promote health benefits in cattle. While the data reported herein, only show trends for the impact of probiotics on alpha diversity, we recognize that we have a relatively small sample size (*n* = 10 calves per treatment) and further research is needed to determine if probiotics may influence alpha diversity in larger sample sizes.

In terms of beta diversity, significant spatial variability in microbial taxa composition was observed among samples across all anatomical locations. This indicates distinct differences in bacterial profiles between nostril-lung, tonsil-lung, and nostril-tonsil sample pairs, while statistical analysis suggested the oral probiotic appeared to impact overall beta diversity. Taken together, the lung samples exhibited significantly different alpha and beta diversity compared to both nostril and tonsil samples, consistent with previous studies (McDaneld et al., 2024). This suggests that the lung microbiome represents a unique microbial community distinct from the calf’s upper respiratory tract, highlighting the importance of considering anatomical (spatial) specificity in microbiome studies.

The bacterial taxa profiles of the nostril, tonsil, and lung samples showed a degree of consistency with previous studies. The genera *Corynebacterium, Prevotella, Stenotrophomonas, Neisseria,* and *Alysiella* were also reported to be present in the upper respiratory tract by Centeno-Martinez et al. (2022), McDaneld et al. (2018), and Huffnagle et al. (2017). While the genera *Elizabethkingia*, *Flavobacterium, Aeromicrobium* and *Prevotella* were also reported to be present in the lower respiratory tract by Hu et al. (2017) and McDaneld et al. (2024). Several of these bacterial taxa have been previously reported to be present in commensal bacterial populations of the respiratory tract and have a role in overall health. Uddin et al. (2023) reported that abundance of the genus *Corynebacterium* changed during transportation of beef cattle and was associated with blood cortisol concentrations. While Timsit et al. (2018) identified *Corynebacterium* in the commensal bacterial populations of the upper respiratory tract of both healthy cattle and cattle diagnosed with bronchopneumonia. *Prevotella* has been predominantly associated with the commensal population of the gut; however, recent studies have identified *Prevotella* in populations of the upper respiratory tract including the tonsil and lungs (Raabis et al., 2022). Overall, it is important to note the presence of variations in the bacterial composition and abundances among the anatomical locations (nostril, tonsil, and lung) while some genera are shared.

While evaluating bacterial pathogen profiles promises to accelerate the understanding of how the overall microbiome profiles associate with and/or are involved with promoting disease, there is still much to be learned about individual pathogens known to cause disease in animals. The most common bacterial organisms identified in animals that succumb to BRD are *Mannheimia haemolytcia*, *Pasteurella multocida*, *Histophilus somni*, and *Mycoplasma bovis*. However, these same bacterial species have also been found in apparently healthy, unaffected cattle. For the data presented herein, *Mycoplasma* was present in all three sampling sites. *Mannheimia* and *Pasteurella* were present in nostril and tonsil sampling sties, while *Histophilus* was only present in the nostril samples. It is important to note that these genera were at low relative abundance (<10%) for all the sampling sites, and that this low relative abundance may be associated with the calve not being diagnosed with BRD and being of young age (<60 days of age at conclusion of study). Moreover, recent studies have shown that potential pathogens previously assumed to have only a minor role in BRD pathogenesis have become much more prevalent and influential (Murray et al., 2016a; Murray et al., 2016b; Johnston et al., 2017).

The identification of differentially abundant taxa among specific anatomical sites provided insights into how feeding probiotics affects the local microbial environment along the respiratory tract and suggests the abundance of these taxa shifts rapidly in the overall microbial composition. While there were minimal overlaps between differentially abundant taxa among each respiratory tract site and across sampling times, there were taxa present in more than one site of the respiratory tract. This overlap has been observed in other studies (Nicola et al., 2017; Zeineldin et al., 2017; Timsit et al., 2018; Howe et al., 2023).

Interestingly, the only genus that is common in lung, nostril and tonsil samples and differentially abundant in all three sampling sites is *Finegoldia*, which is more abundant in treated samples compared to control in all three niches. The sole species within this genus, *Finegoldia magna*, has been reported as an opportunistic pathogen, and is associated with bacterial vaginosis (Murphy and Frick, 2013) and may promote eczema if found in the human gut microbiota (Cheung et al., 2023). In cattle, an increased prevalence of this genus was observed in the vagina of dairy cows with purulent vaginal discharge postpartum (Moore et al., 2023). Additional research will be needed to further elucidate the association between probiotic treatment and the prevalence of *Finegoldia* in the respiratory tract.

Moreover, some of the differentially abundant taxa identified in lung, nostril, and tonsil samples have been reported to be part of the oral microbiota, such as *Pseudomonas, Burkholderia*, and *Actinobacteria*, which were found in the mouth of healthy cattle (Borsanelli et al., 2018). The same study also demonstrated that *Prevotella* and *Fusobacterium* appeared to be differentially abundant in cattle with an infection. *Pasteurellaceae*, *Moraxellaceae*, and *Neisseriaceae* have been detected in the oral cavity of calves (Barden et al., 2020). Members of the *Pasteurellaceae* family have been associated with Bovine Respiratory Disease. Recent data have supported a lung-gut axis, as bacterial taxa previously thought to be predominant in the gut have been found in the lung and vice versa (Glendinning et al., 2017), resulting in the microbiome of one location having the potential to influence the microbiome of the other location.

## Conclusion

In conclusion, our study revealed variations in bacterial diversity within the nostril, tonsil, and lung samples, indicating distinct microbial compositions between upper and lower respiratory tract sites. This observance across anatomical locations, highlights the importance of anatomical specificity in microbiome studies. Furthermore, the identification of differentially abundant taxa provided insights into the impact of probiotic treatments on the respiratory tract microbiome. These findings contribute to the understanding of the dynamic nature of bacterial diversity and the potential probiotic effects within the bovine respiratory tract and may guide future studies on animal health, disease prevention, and management.

## Author’s note

Mention of trade names or commercial products in this publication is solely for the purpose of providing specific information and does not imply recommendation or endorsement by the U.S. Department of Agriculture. USDA is an equal opportunity provider and employer.

## Data Availability

The datasets presented in this study can be found in online repositories. The names of the repository/repositories and accession number(s) can be found at: https://www.ncbi.nlm.nih.gov/, PRJNA1148230; https://www.ncbi.nlm.nih.gov/, PRJNA1018539.

## Footnotes

^1^ https://ftp.microbio.me/greengenes_release/current/2022.10.backbone.full-length.fna.qza
