# Supplementary material for: Insights into microbial compositions of the respiratory tract of neonatal dairy calves in a longitudinal probiotic trial through 16S rRNA sequencing
Source: Front Microbiol. 2025 Jan 8;15:1499531. doi: 10.3389/fmicb.2024.1499531 (PMC11751226; doi:10.3389/fmicb.2024.1499531)
Supplement: Supplementary file 1 [file Data_Sheet_1.pdf]

A.

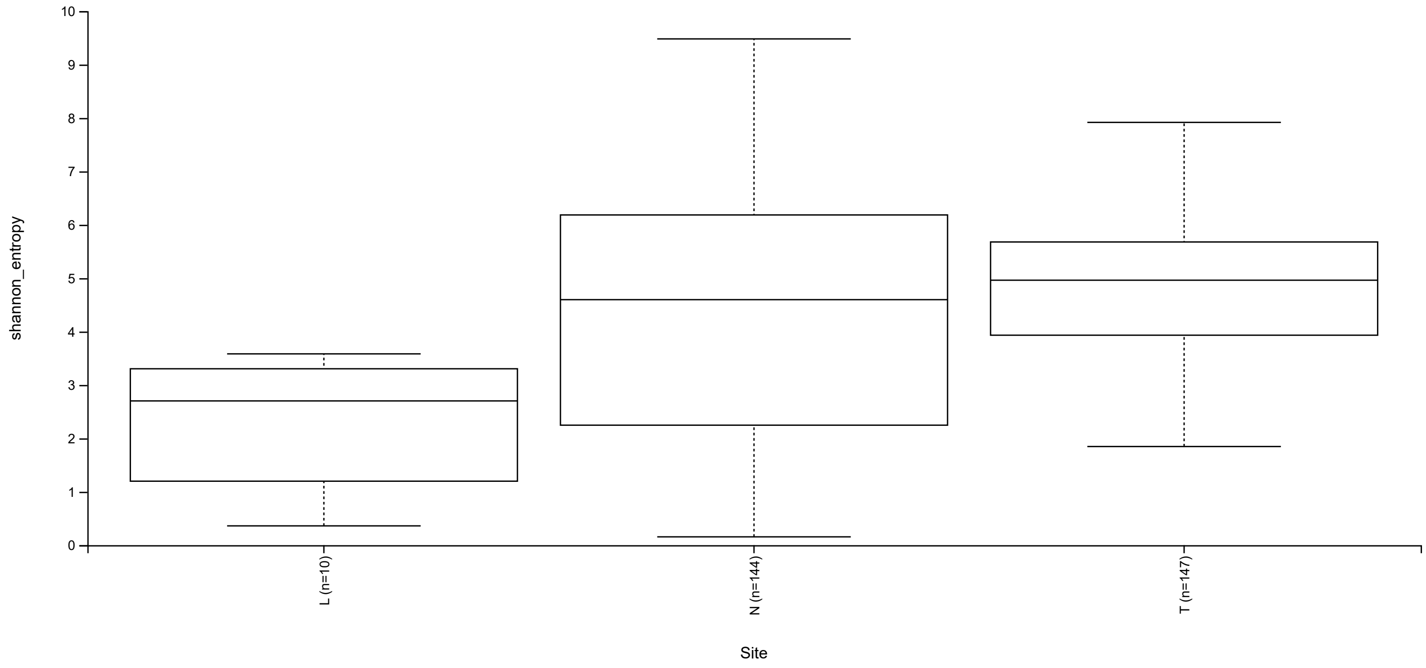

B.

Group 1	Group 2	H	p-value	q-value
L (n=10)	N (n=144)	7.969086022	0.004758296	0.007137444
L (n=10)	T (n=147)	20.44128132	0.000006149	0.000018447
N (n=144)	T (n=147)	1.675521464	0.195520609	0.195520609

Supplemental Figure 1. Boxplot and Kruskal-Wallis Pairwise Comparison for Shannon Diversity Index between different anatomical sites (A) Boxplot of Shannon Diversity Index for each site (B) Kruskal-Wallis Pairwise Comparison results.
