# Supplementary material for: Insights into microbial compositions of the respiratory tract of neonatal dairy calves in a longitudinal probiotic trial through 16S rRNA sequencing
Source: Front Microbiol. 2025 Jan 8;15:1499531. doi: 10.3389/fmicb.2024.1499531 (PMC11751226; doi:10.3389/fmicb.2024.1499531)
Supplement: Supplementary file 2 [file Data_Sheet_2.pdf]

A.

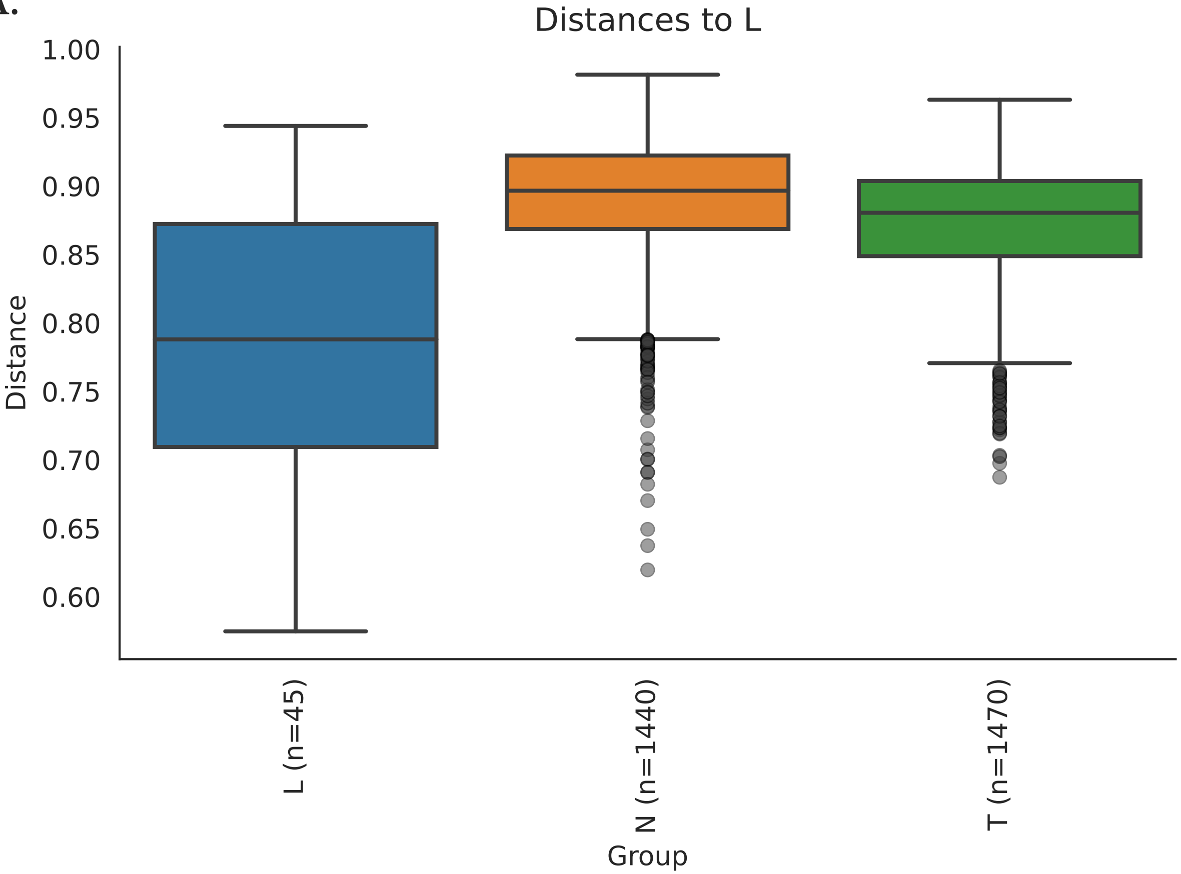

Distances to L
1.00
0.95
0.90
0.85
0.80
0.75
0.70
0.65
0.60
Distance
L (n=45)
N (n=1440)
T (n=1470)
Group

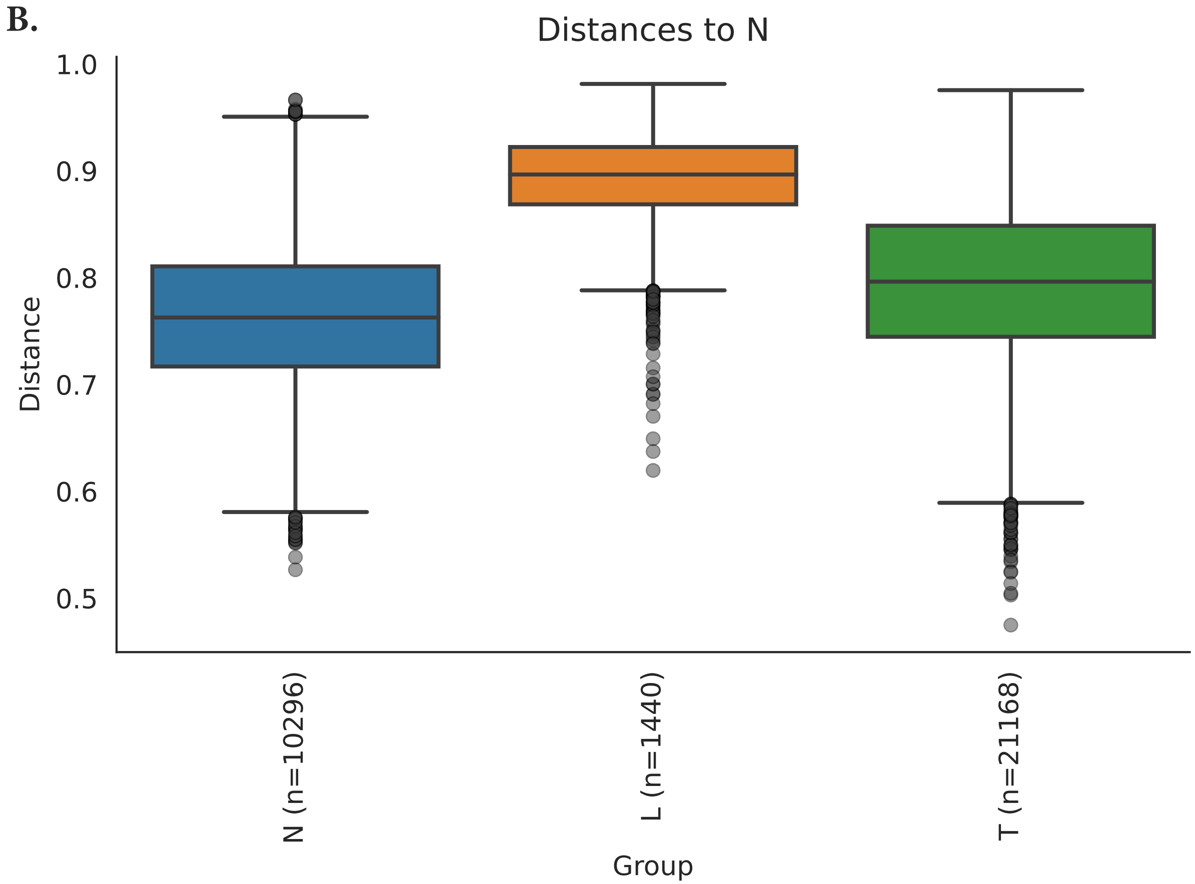

B.
Distances to N
1.0
0.9
0.8
0.7
0.6
0.5
Distance
N (n=10296)
L (n=1440)
T (n=21168)
Group

C.

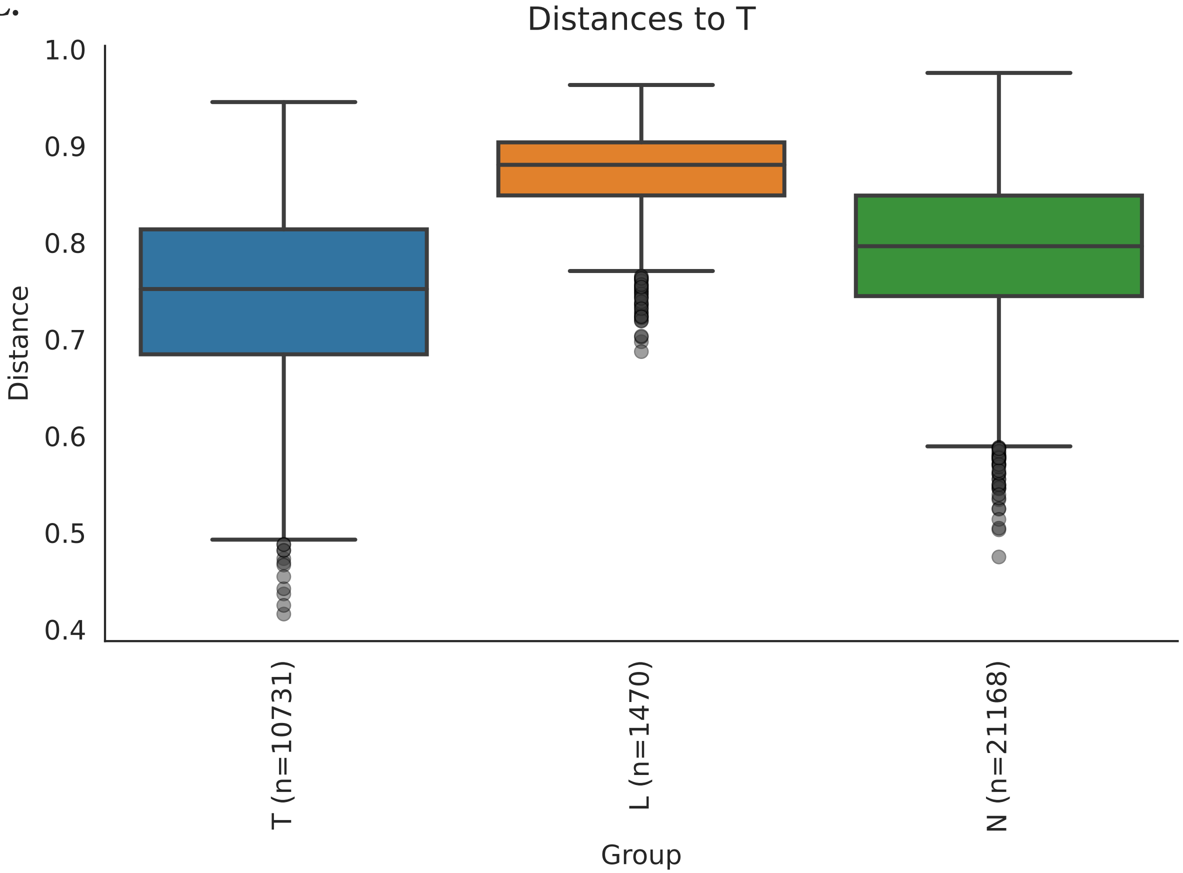
Distances to T
Distance
1.0
0.9
0.8
0.7
0.6
0.5
0.4
T (n=10731)
L (n=1470)
N (n=21168)
Group

D.

Group 1	Group 2	Sample size	Permutations	pseudo-F	p-value	q-value
L	N	154	999	7.04899593	0.001	0.001
L	T	157	999	6.69514814	0.001	0.001
N	T	291	999	16.063584	0.001	0.001

Supplemental Figure 2. Box plot and PERMANOVA test of Unweighted Unifrac Distance between different anatomical sites. (A) Boxplot of unweighted unifrac distance from each site to lung. (B) Boxplot of unweighted unifrac distance from each site to nostril. (C) Boxplot of unweighted unifrac distance from each site to tonsil. (D) PERMANOVA test results.
