# Supplementary material for: Insights into microbial compositions of the respiratory tract of neonatal dairy calves in a longitudinal probiotic trial through 16S rRNA sequencing
Source: Front Microbiol. 2025 Jan 8;15:1499531. doi: 10.3389/fmicb.2024.1499531 (PMC11751226; doi:10.3389/fmicb.2024.1499531)
Supplement: Supplementary file 3 [file Data_Sheet_3.pdf]

A.

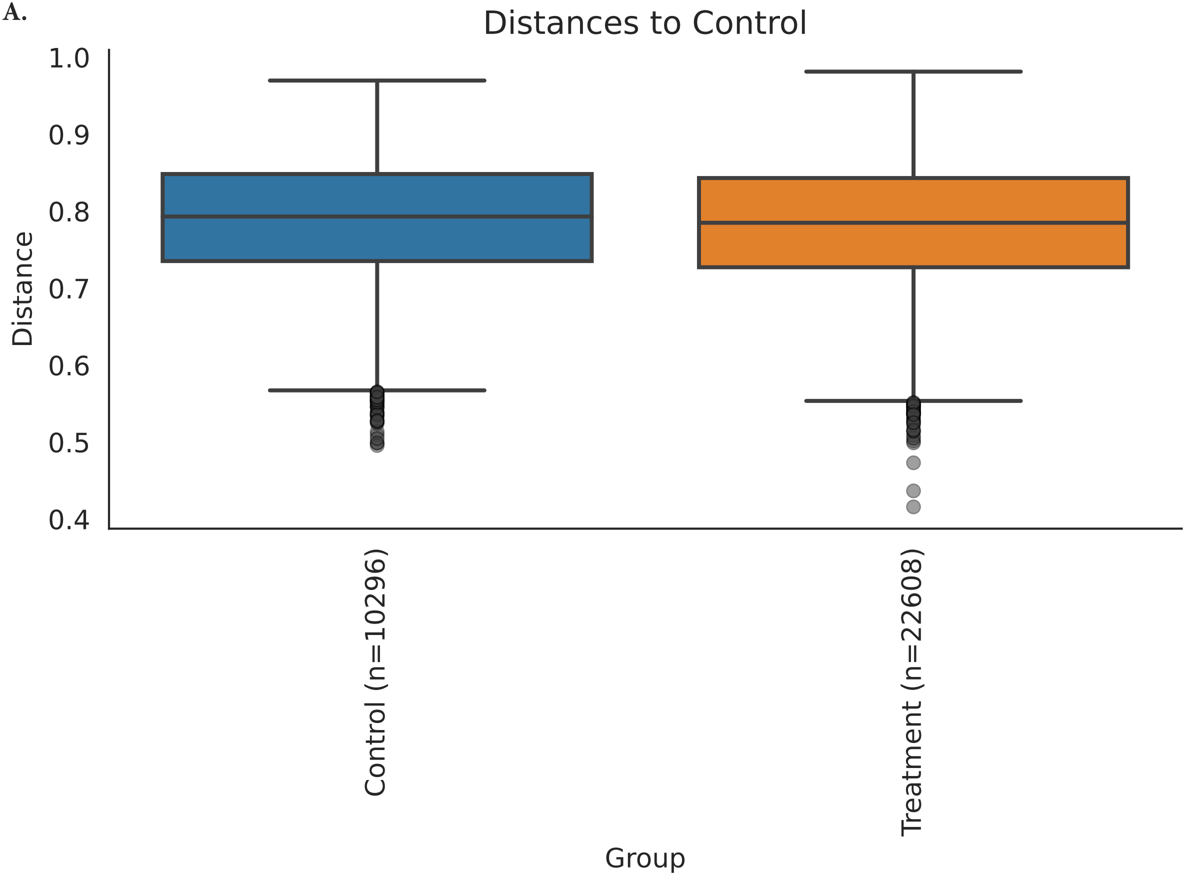
Distances to Control
1.0
0.9
0.8
0.7
0.6
0.5
0.4
Distance
Control (n=10296)
Treatment (n=22608)
Group

B.

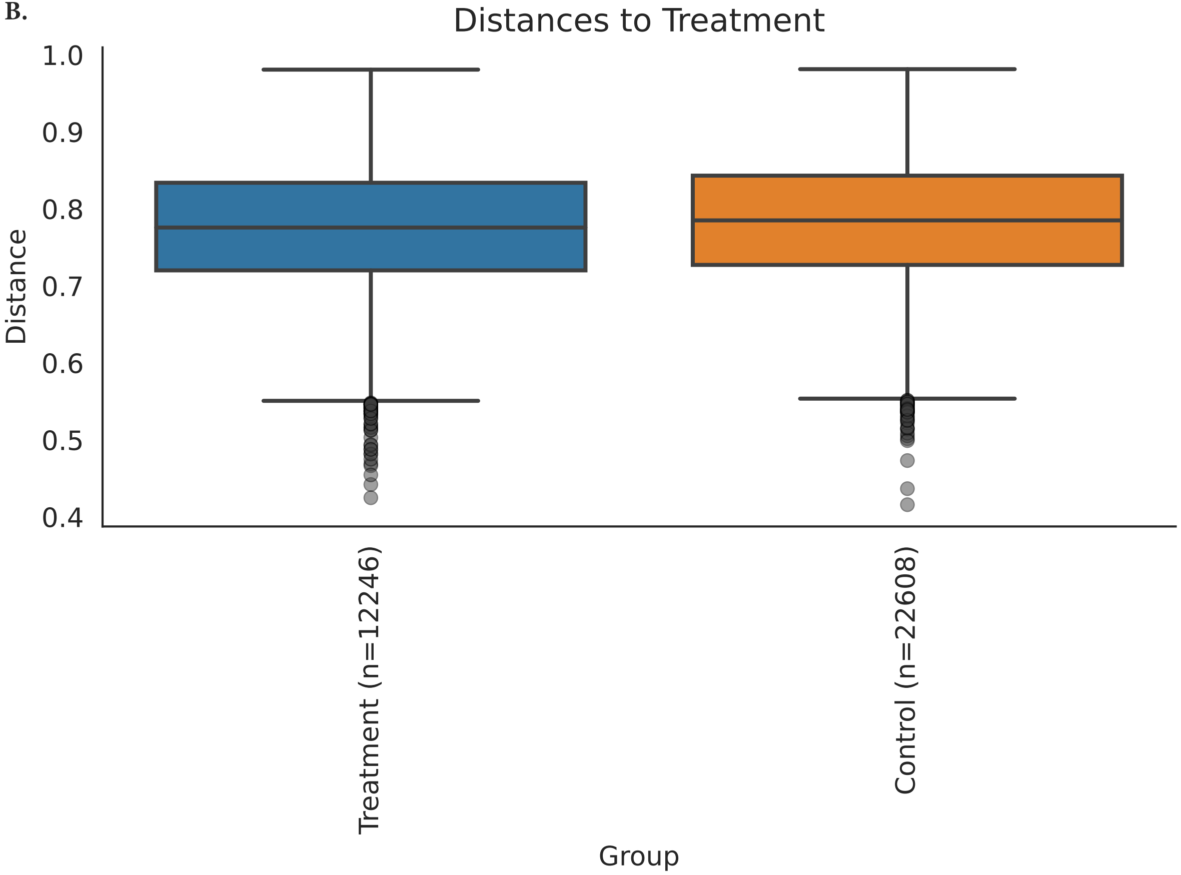
Distances to Treatment
Distance
1.0
0.9
0.8
0.7
0.6
0.5
0.4
Treatment (n=12246)
Control (n=22608)
Group

C.

Group 1	Group 2	Sample size	Permutations	pseudo-F	p-value	q-value
Control	Treatment	301	999	1.406059602	0.041	0.041

Supplemental Figure 3. Box plot and PERMANOVA test of Unweighted Unifrac Distance between treatment and control. (A) Boxplot of unweighted unifrac distance from Treatment to Control. (B) Boxplot of unweighted unifrac distance from Control to Treatment. (C) PERMANOVA test results.
