# Supplementary figures and images for: Insights into microbial compositions of the respiratory tract of neonatal dairy calves in a longitudinal probiotic trial through 16S rRNA sequencing
Source: Front Microbiol. 2025 Jan 8;15:1499531. doi: 10.3389/fmicb.2024.1499531 (PMC11751226; doi:10.3389/fmicb.2024.1499531)

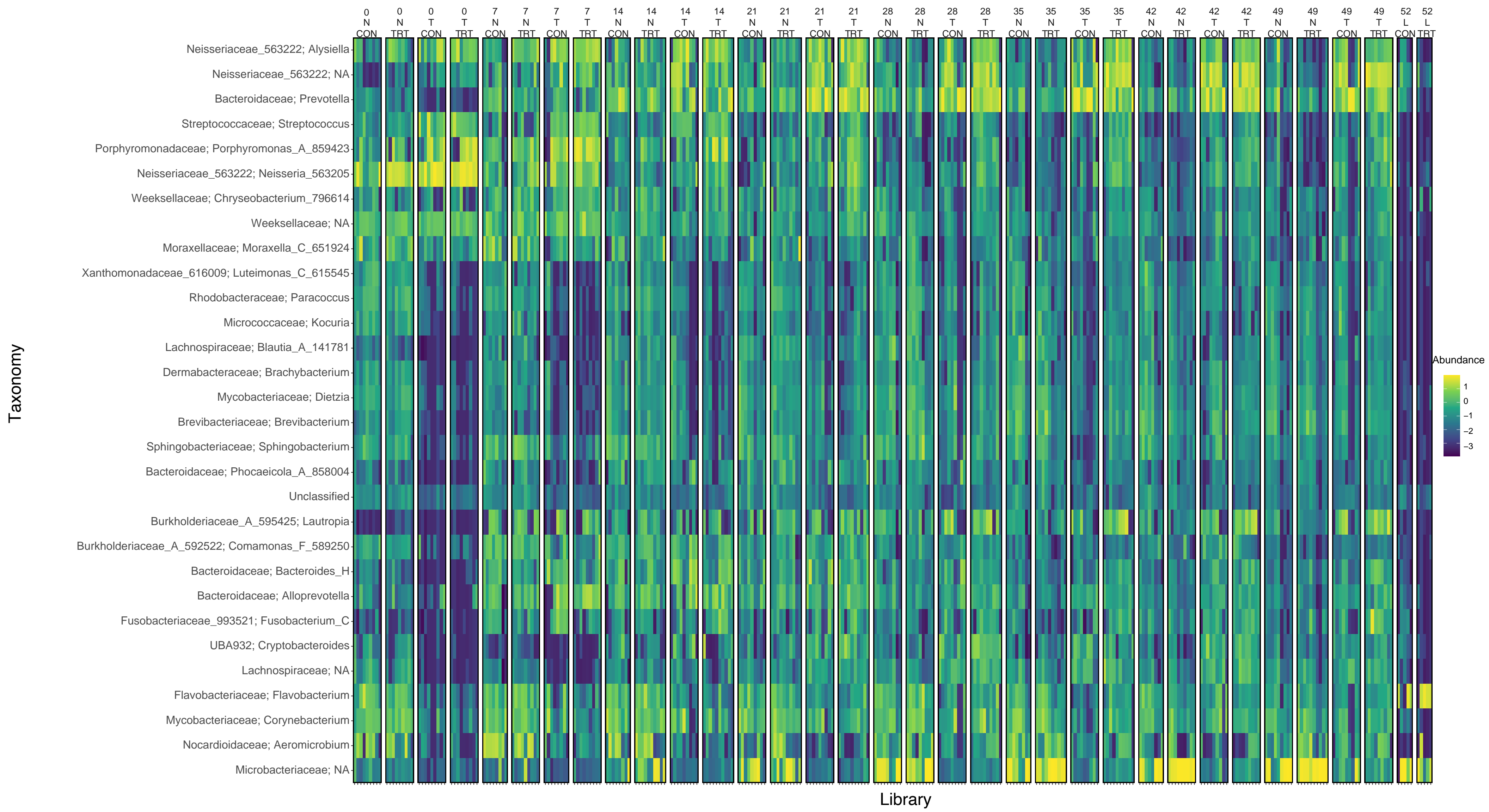

Supplement: Supplementary file 4 [file Data_Sheet_4.pdf]

Nostril Treated vs Control Samples

A

Day 0

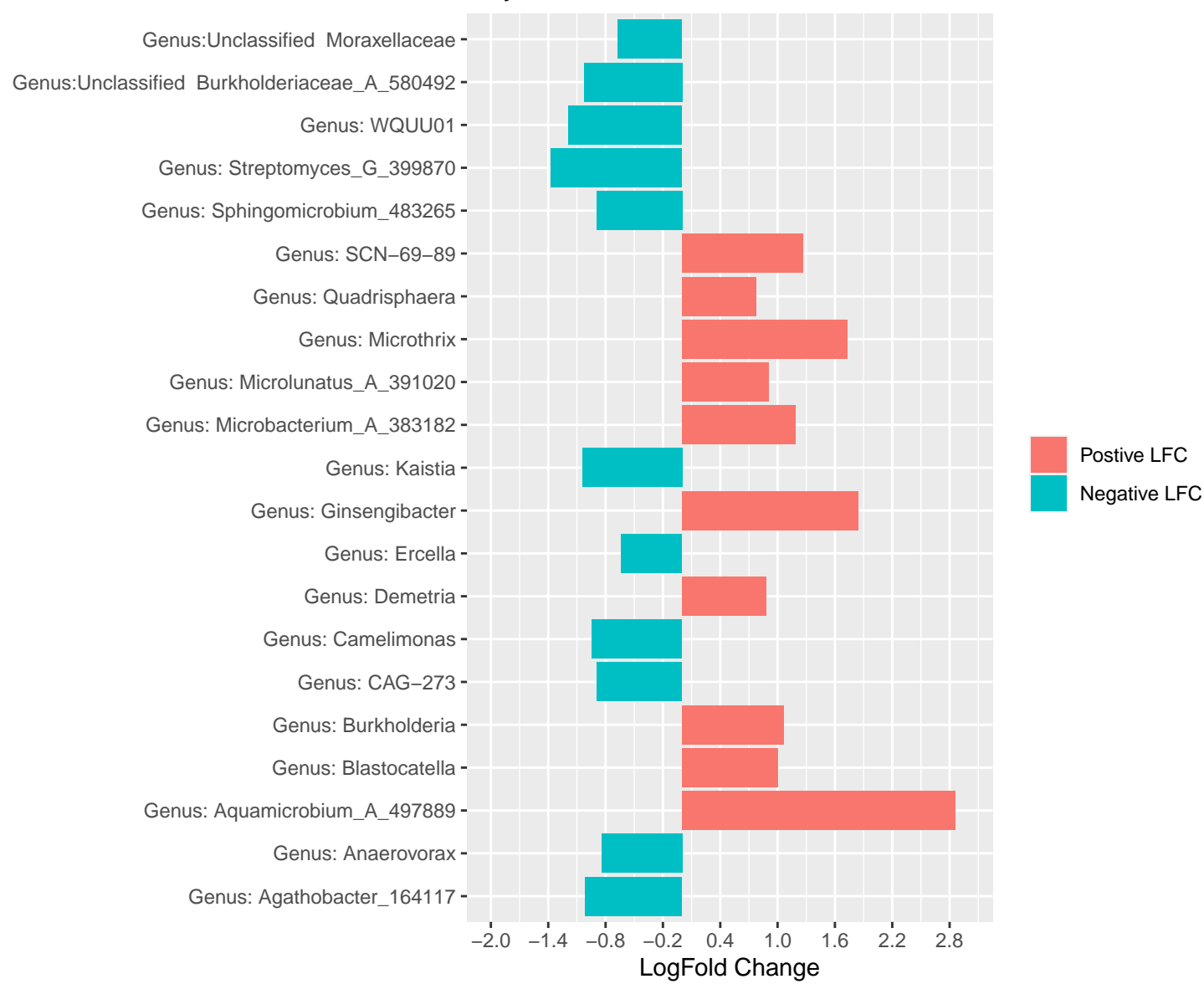

B

Day 7

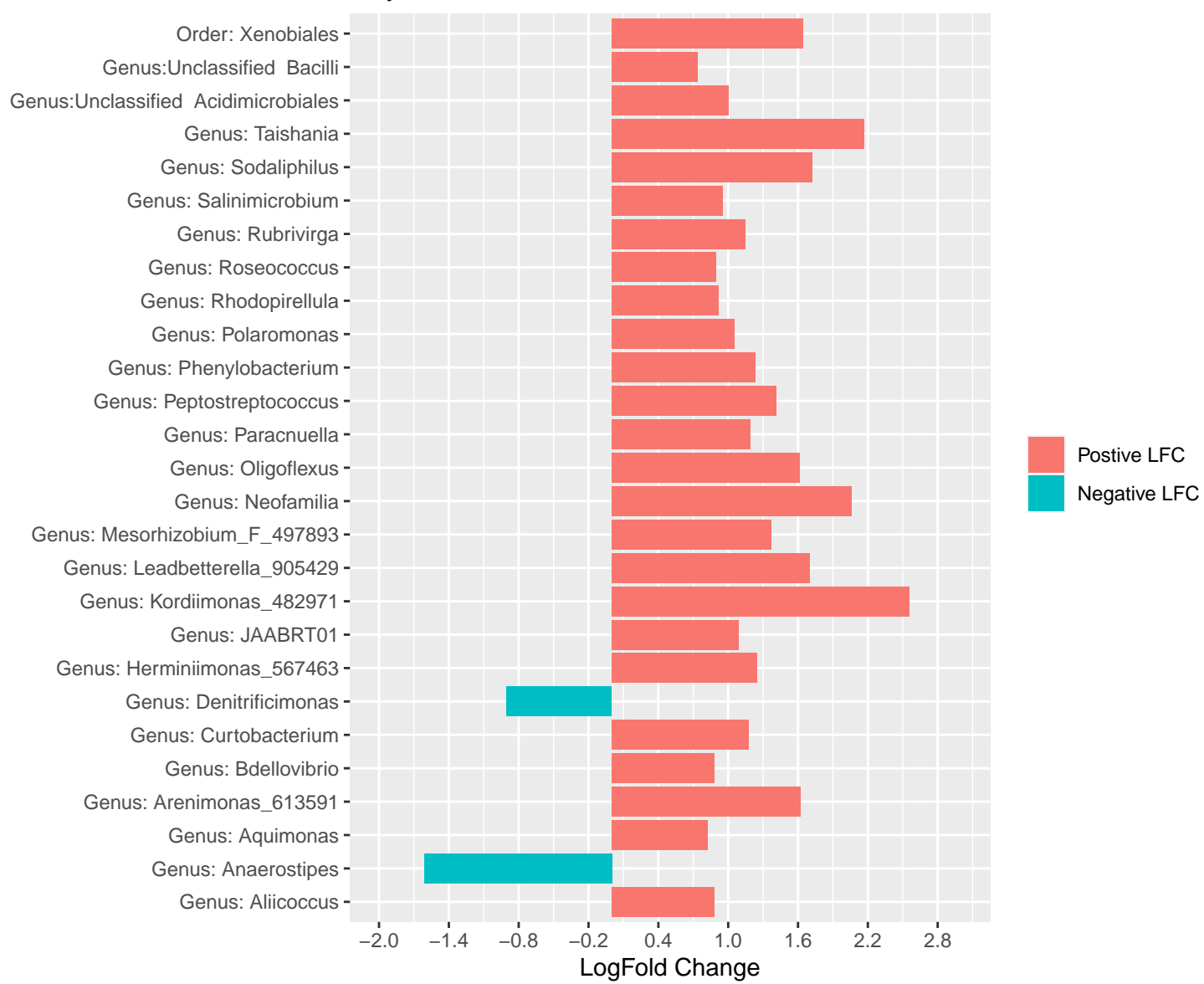

C

Day 14

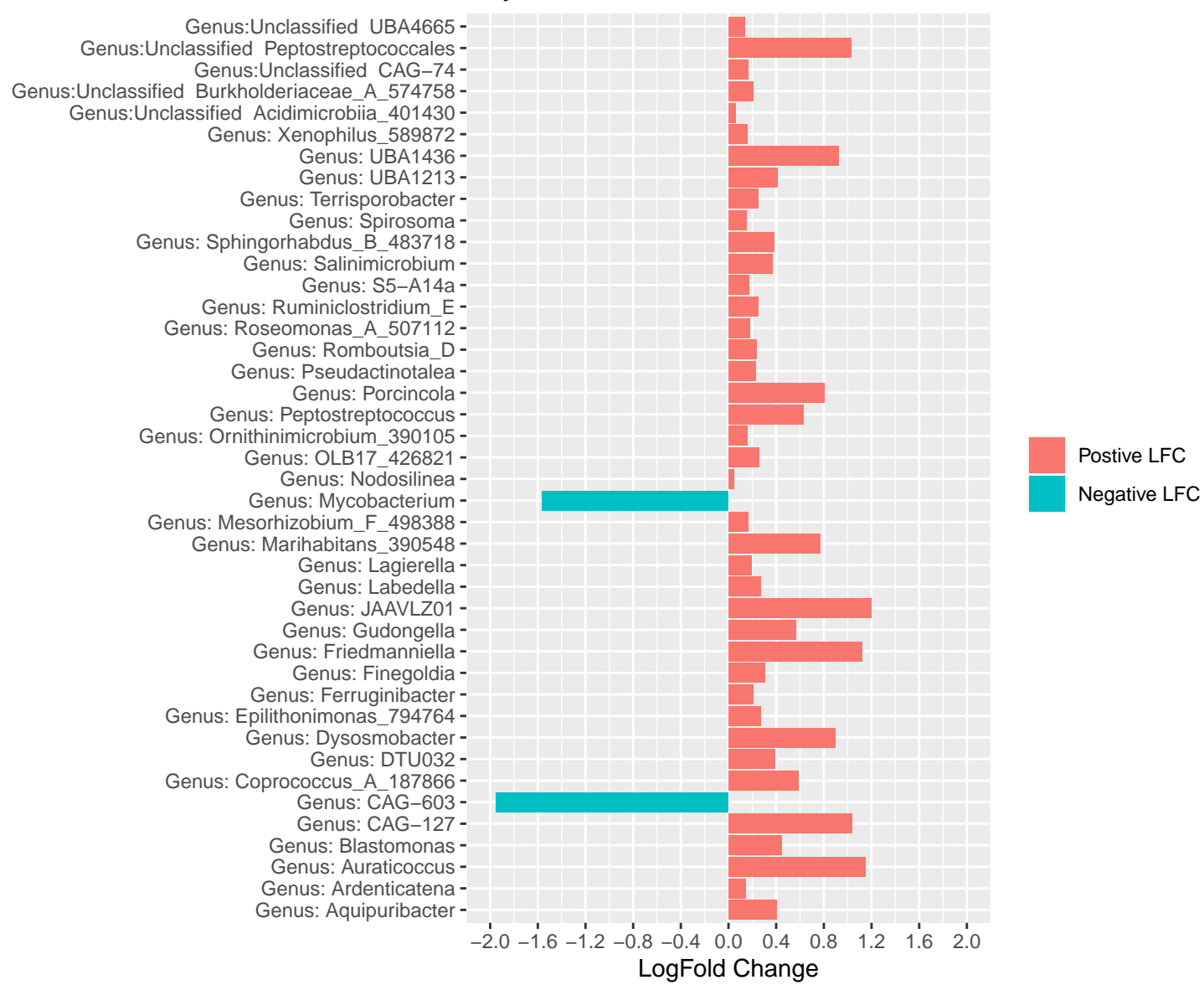

D

Day 21

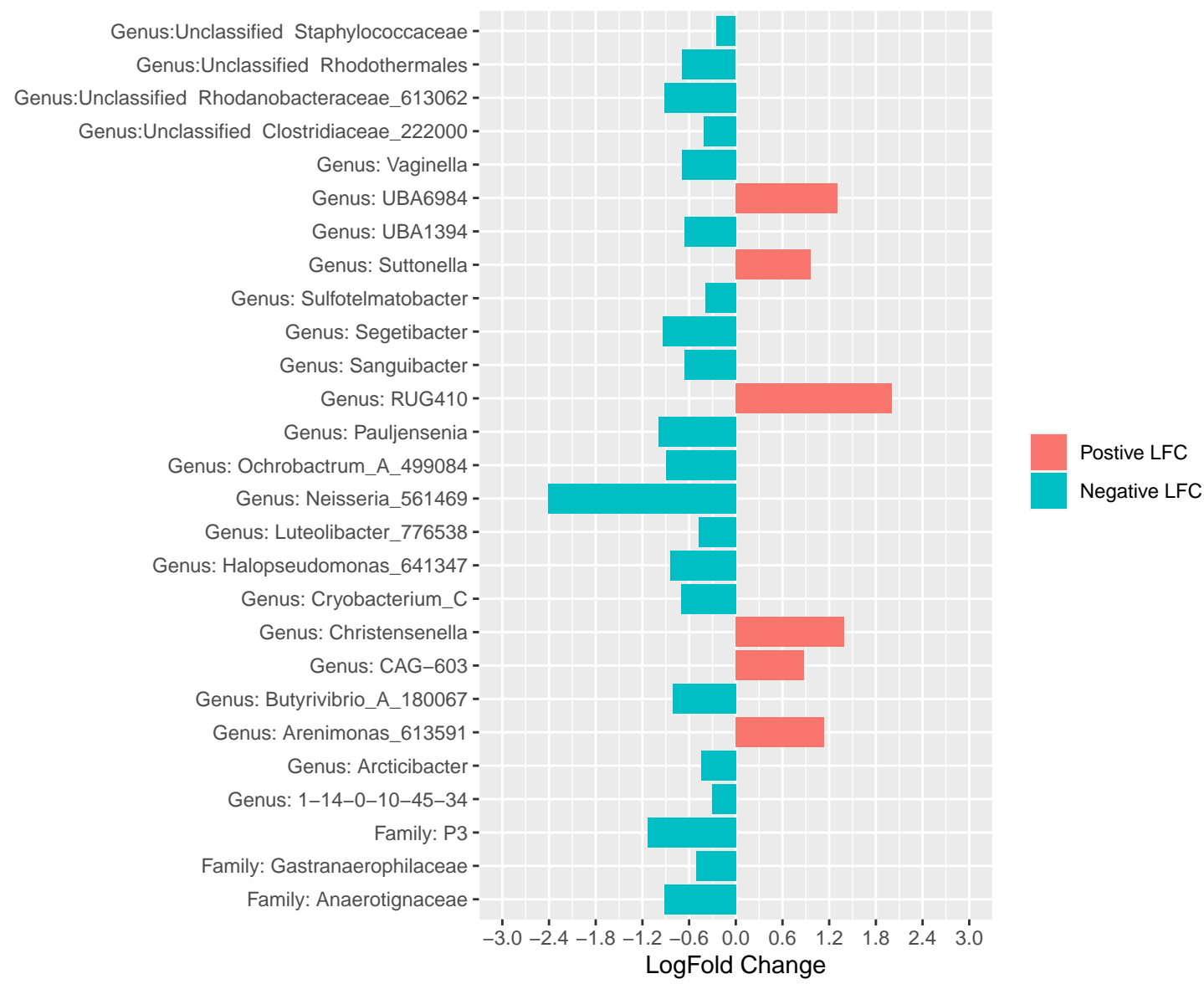

E

Day 28

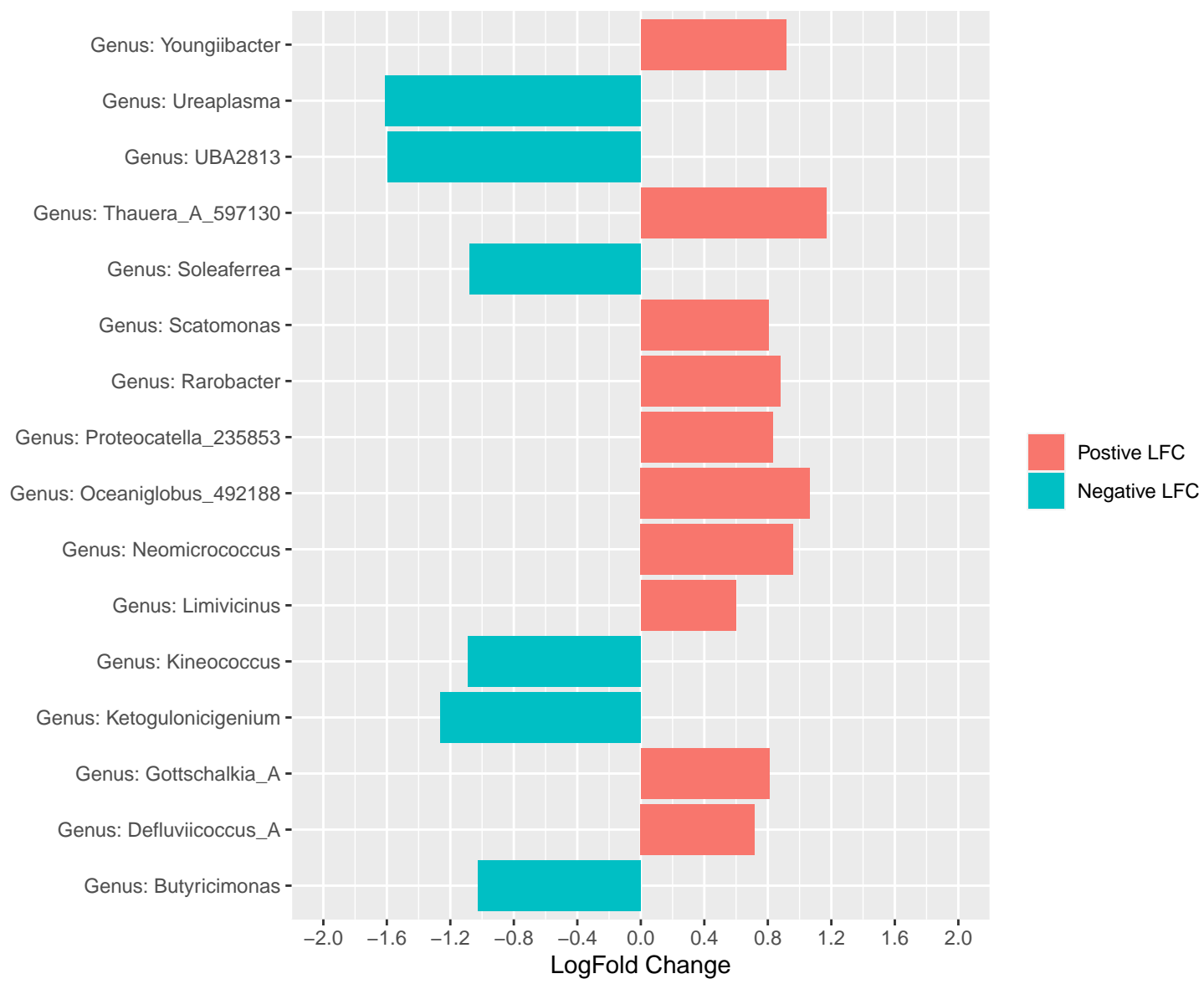

F

Day 35

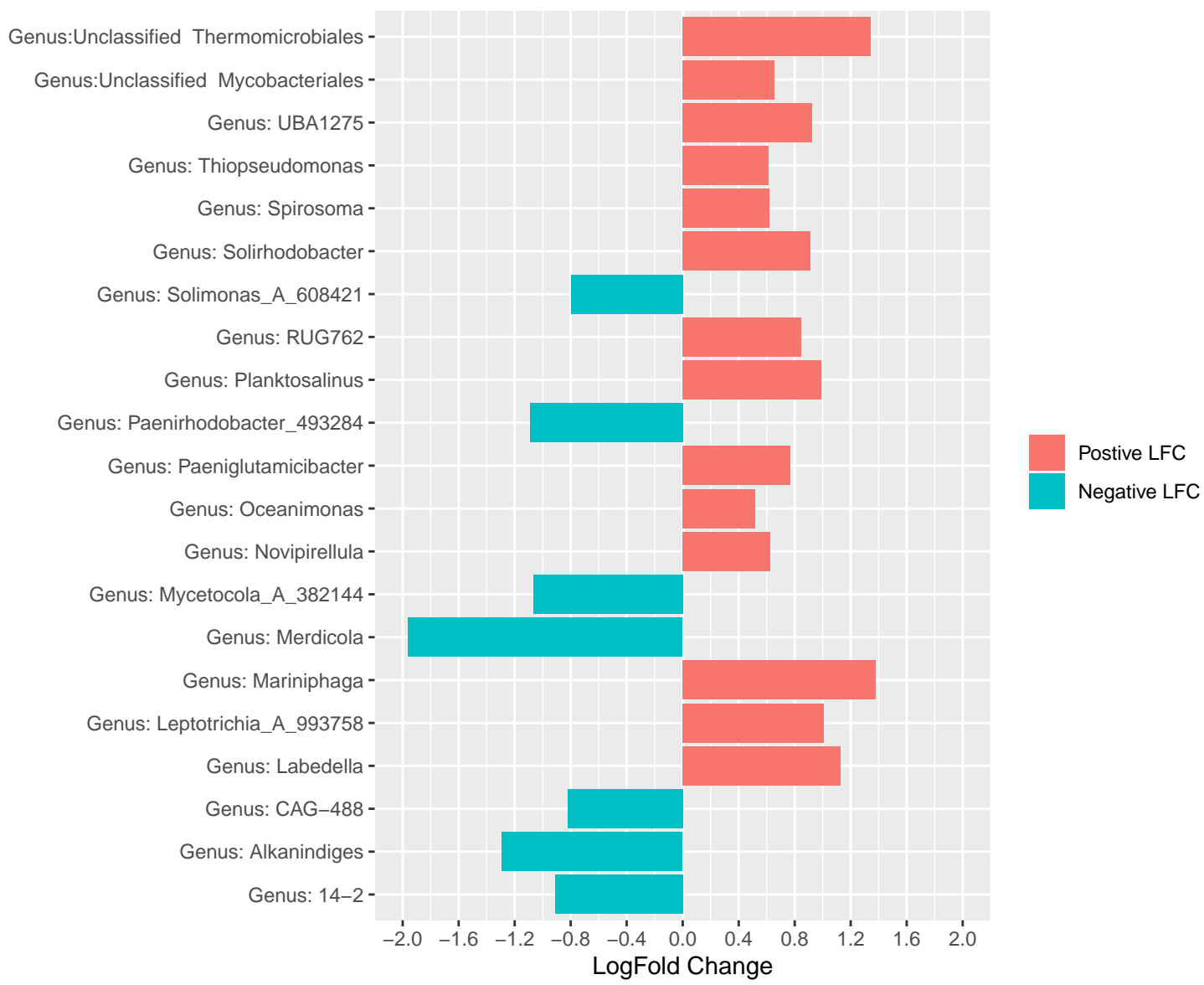

G

Day 42

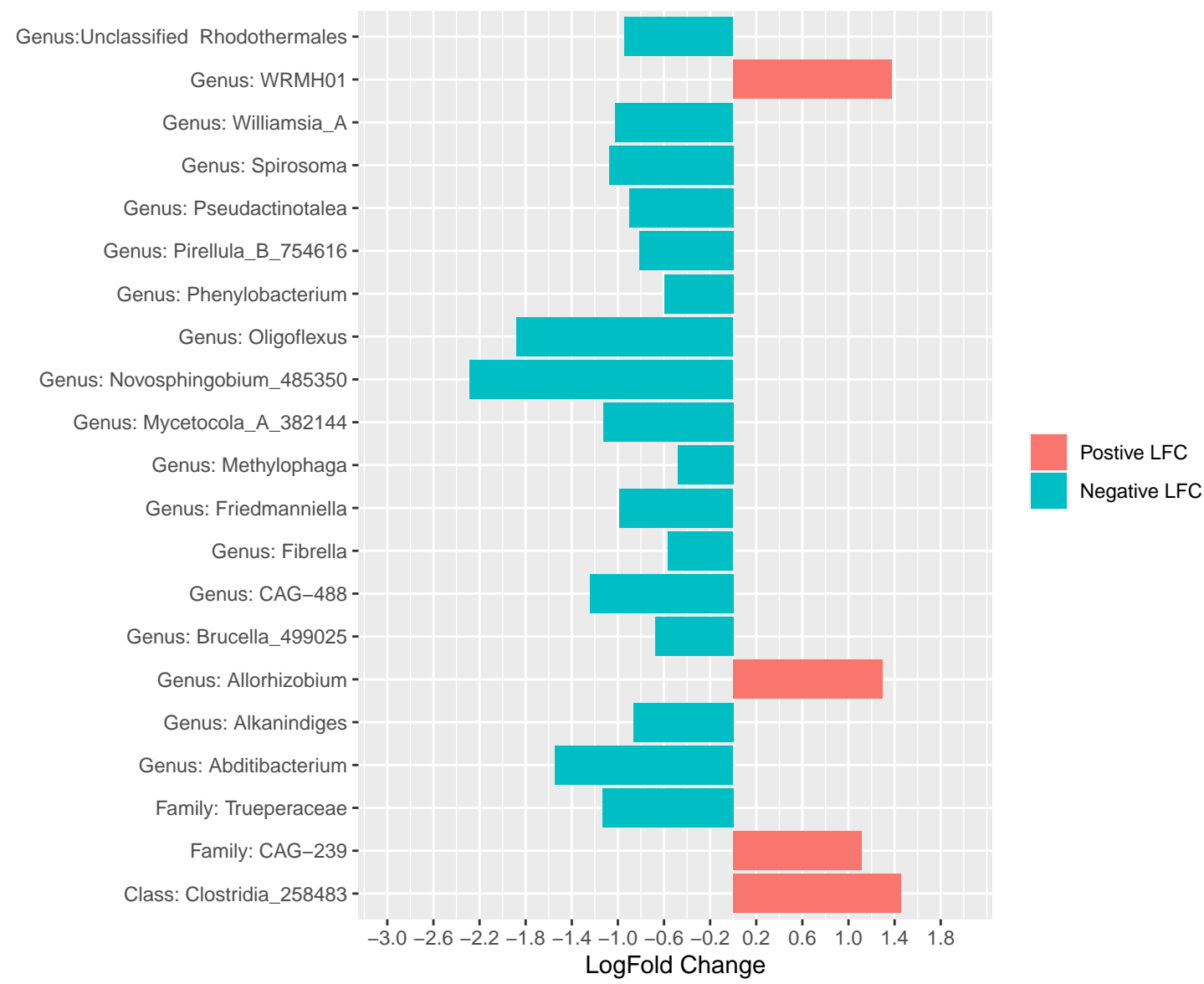

H

Day 49

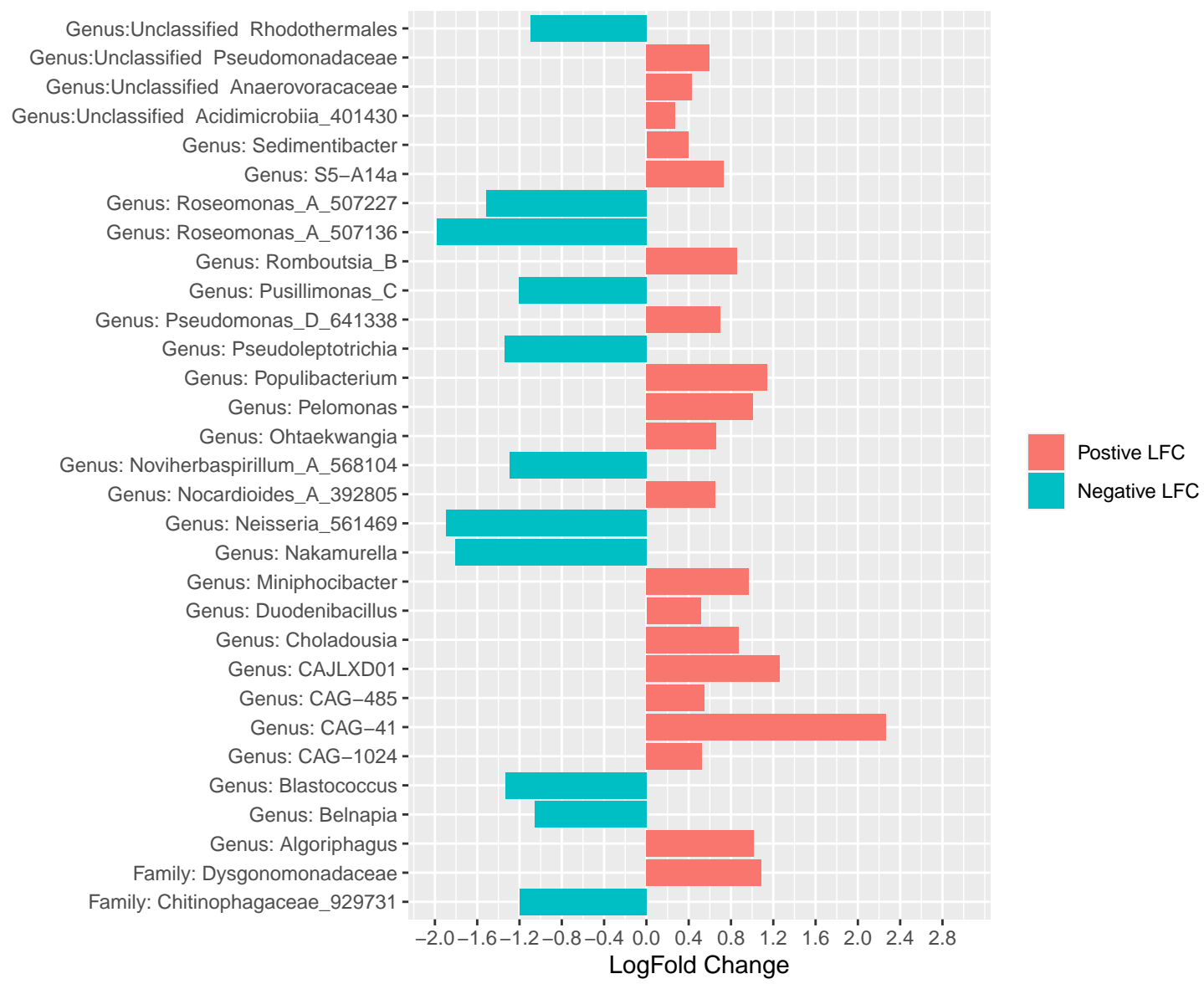

Supplement: Supplementary file 5 [file Data_Sheet_5.pdf]

A

Day 0

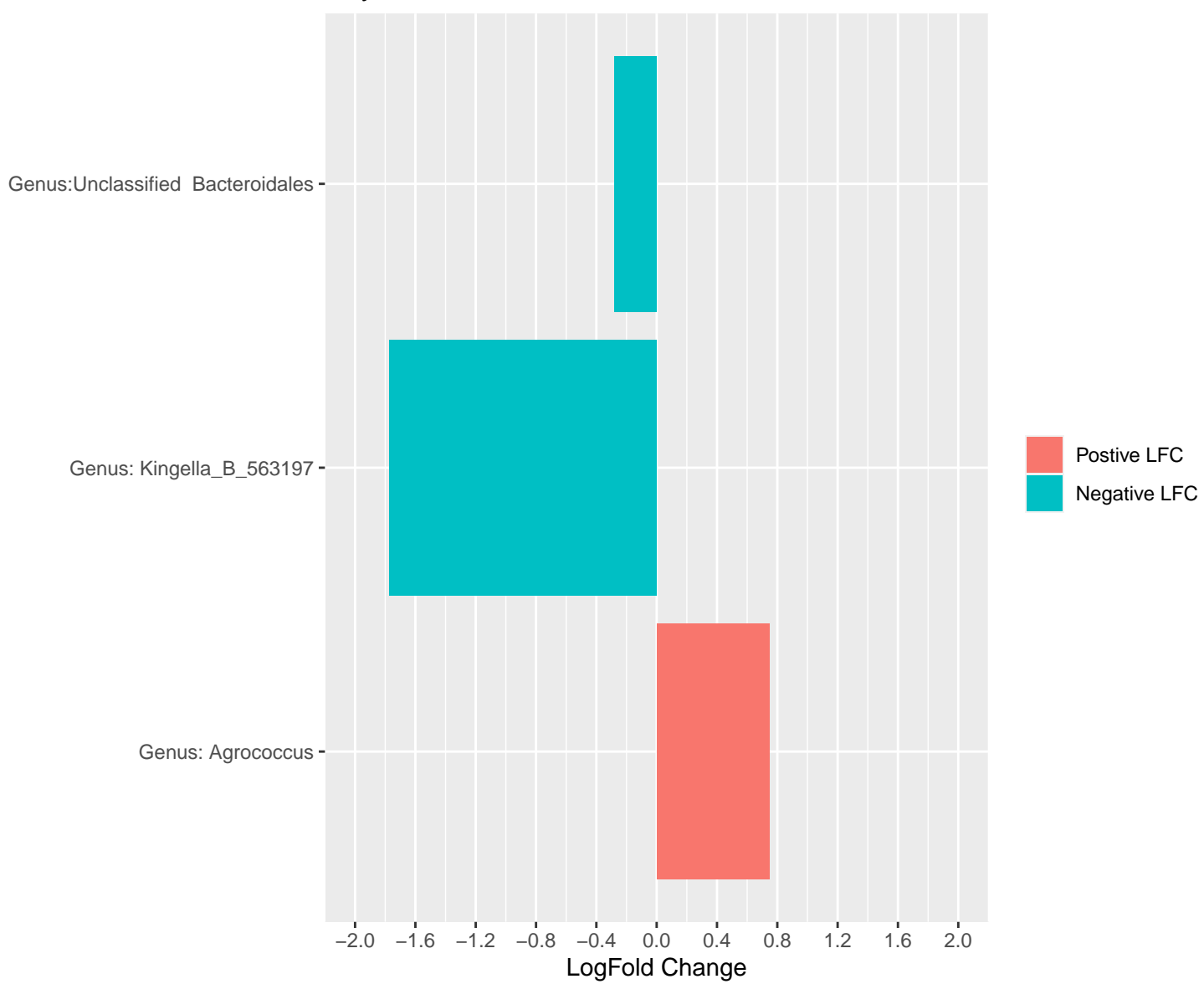

B

Day 7

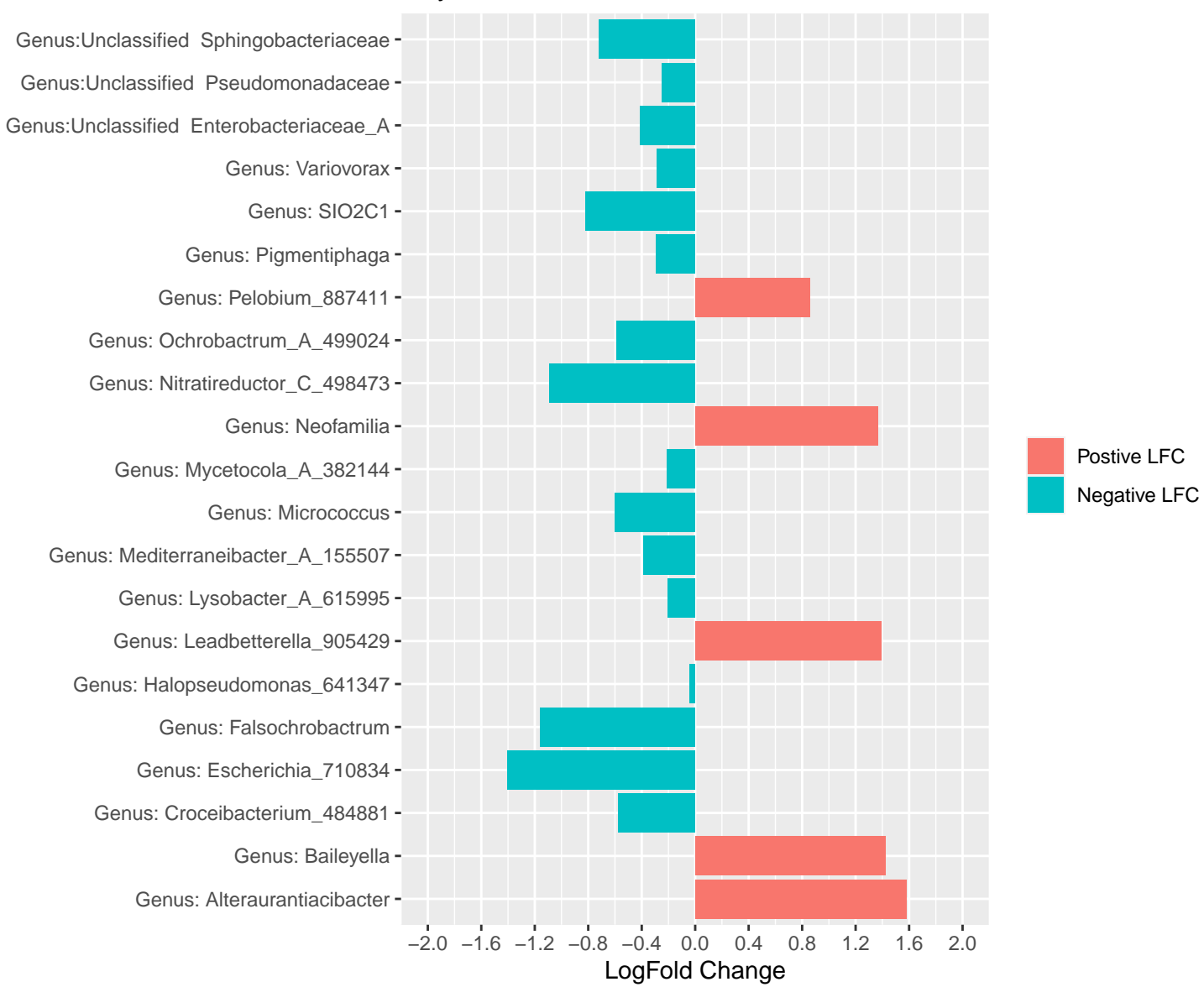

C

Day 14

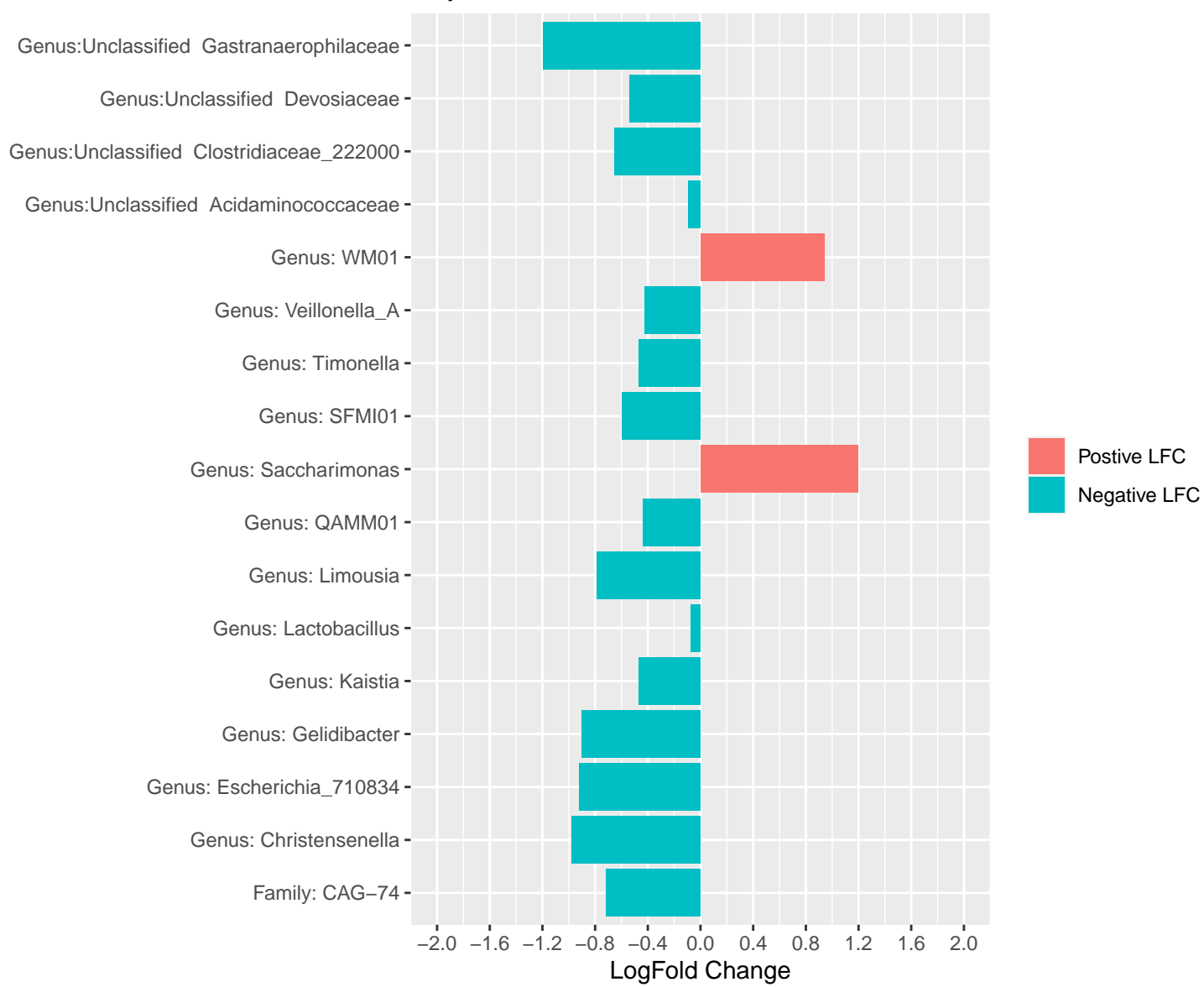

D

Day 21

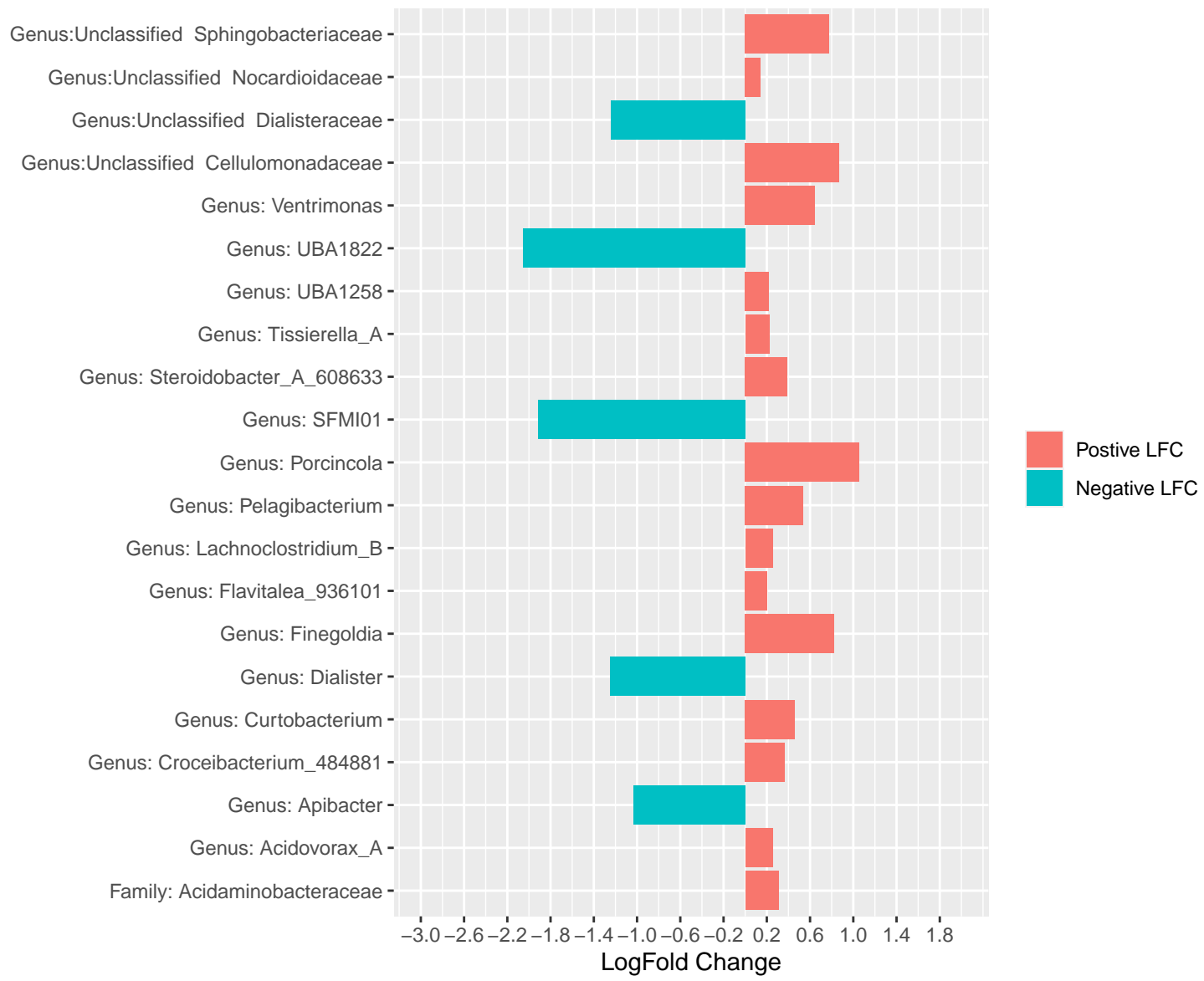

E

Day 28

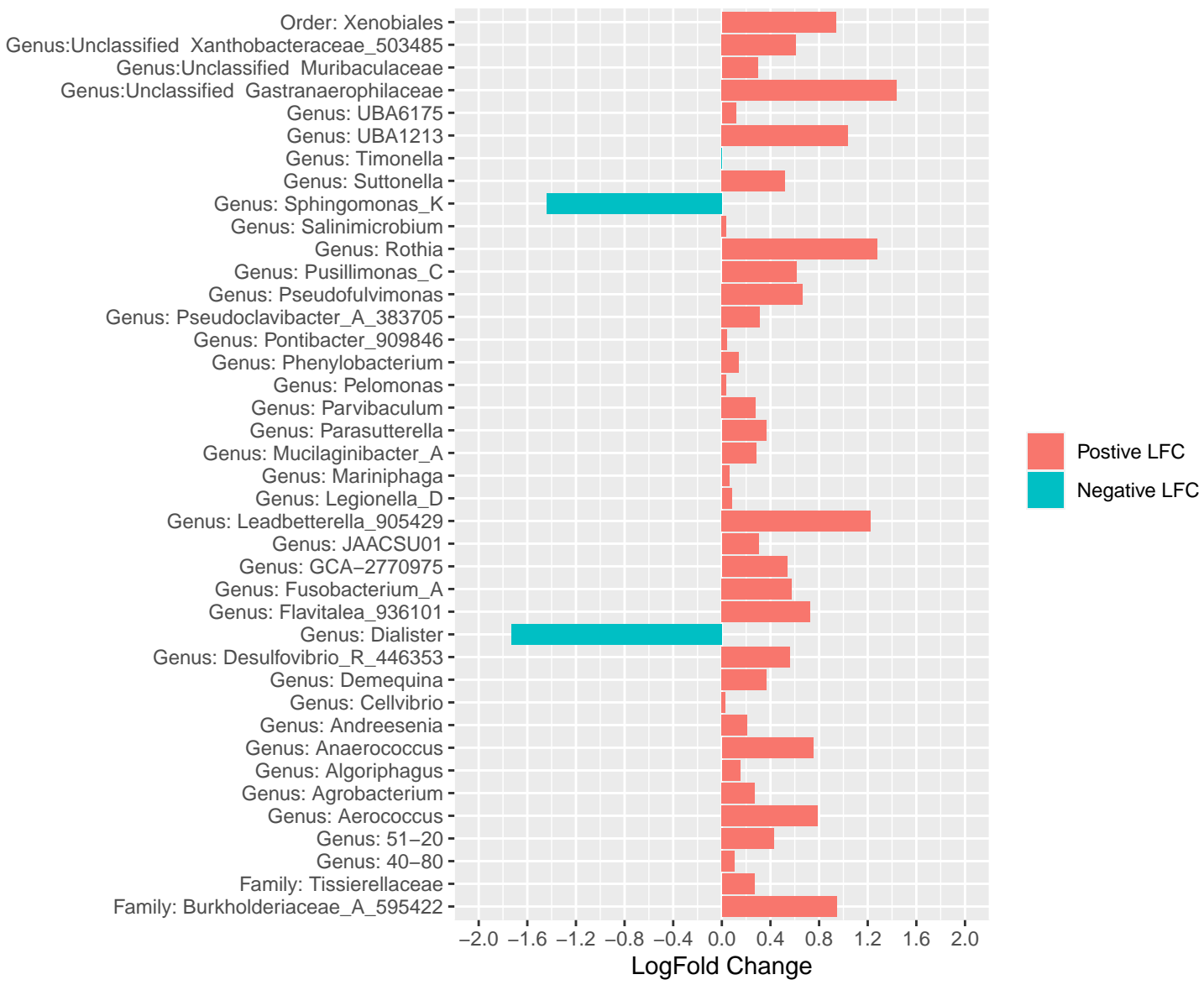

F

Day 35

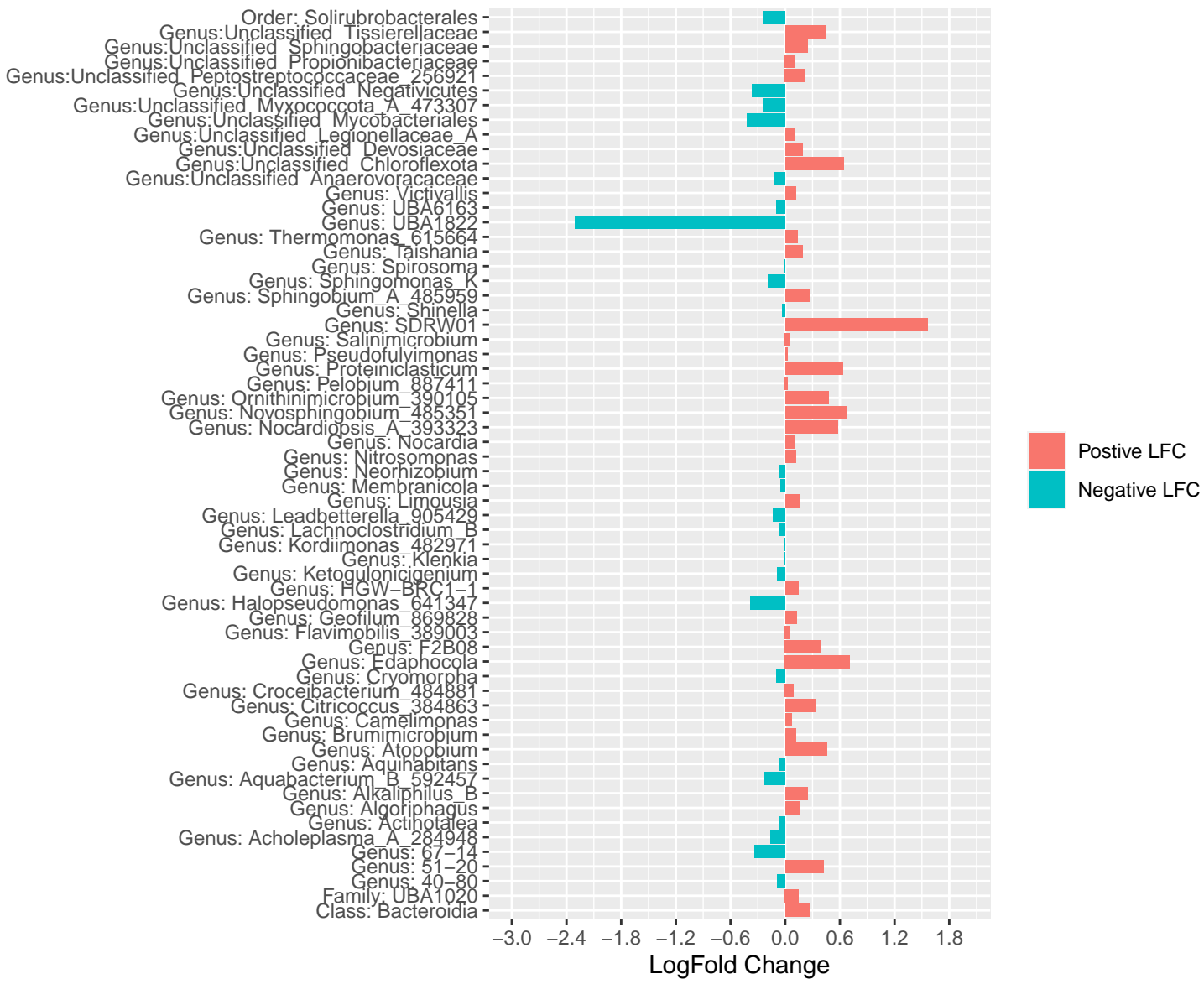

G

Day 42

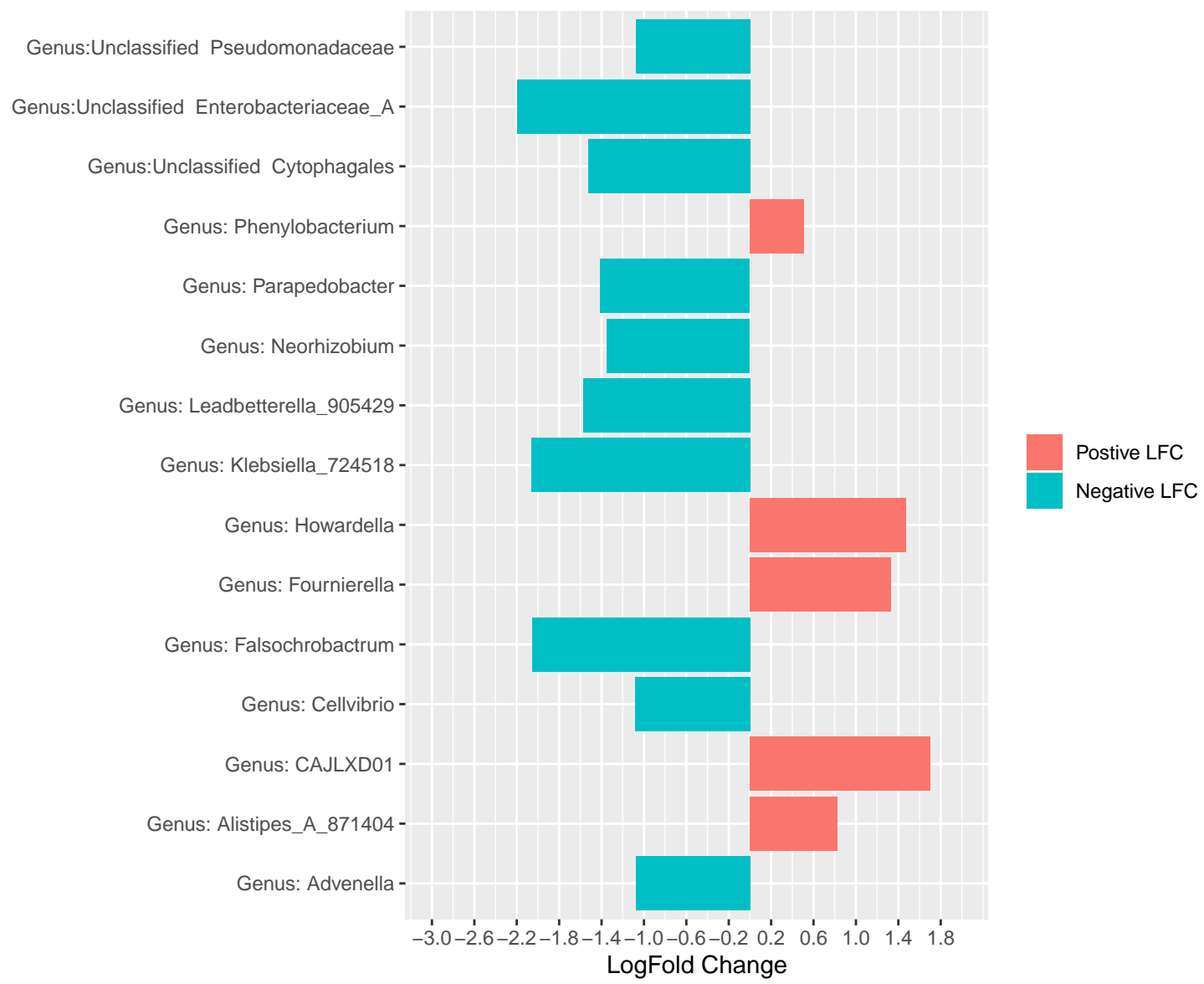

H

Day 49

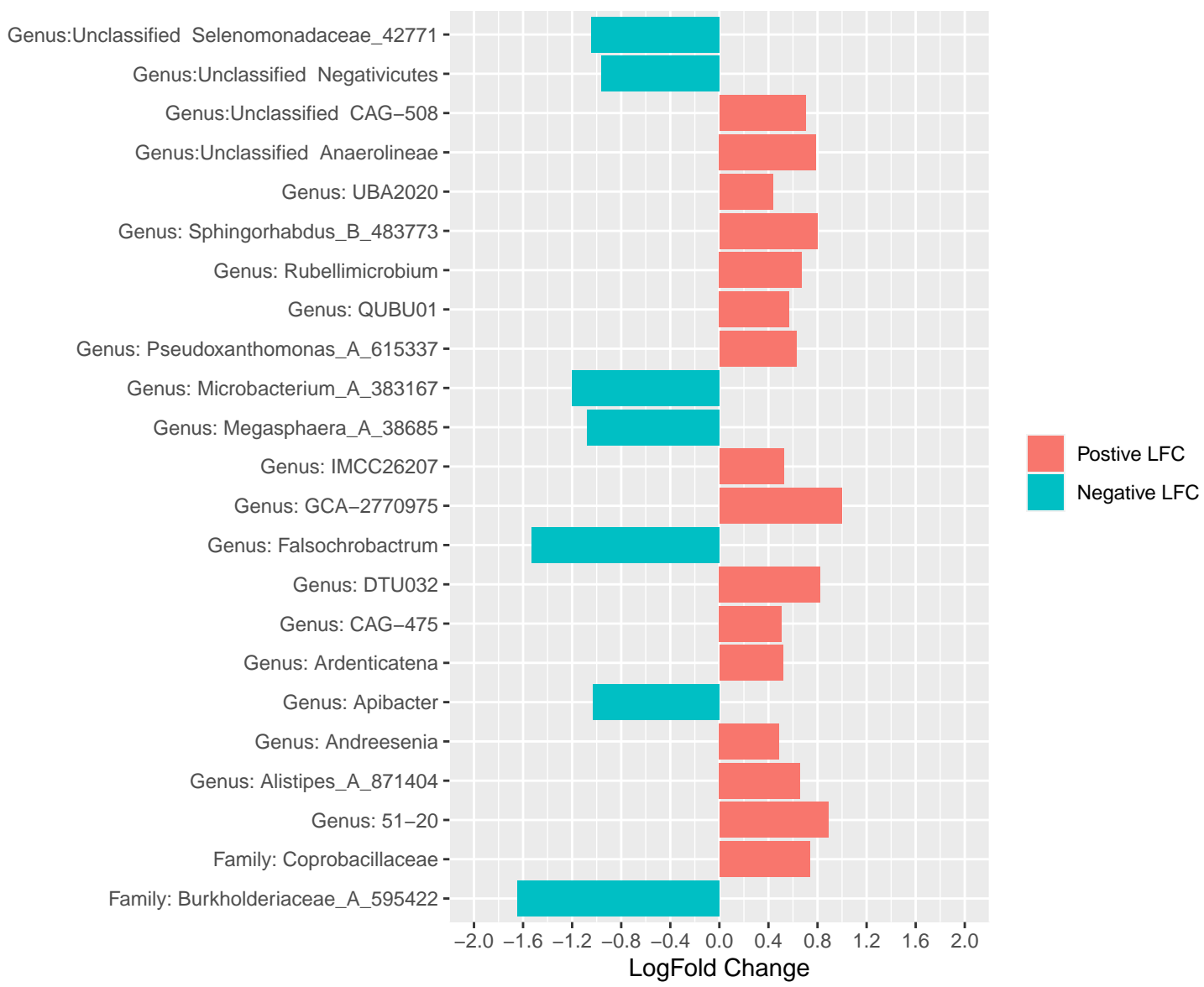

Supplement: Supplementary file 6 [file Data_Sheet_6.pdf]

# Lung Treated vs Control Samples at day 52

Differentially Abundant Genus

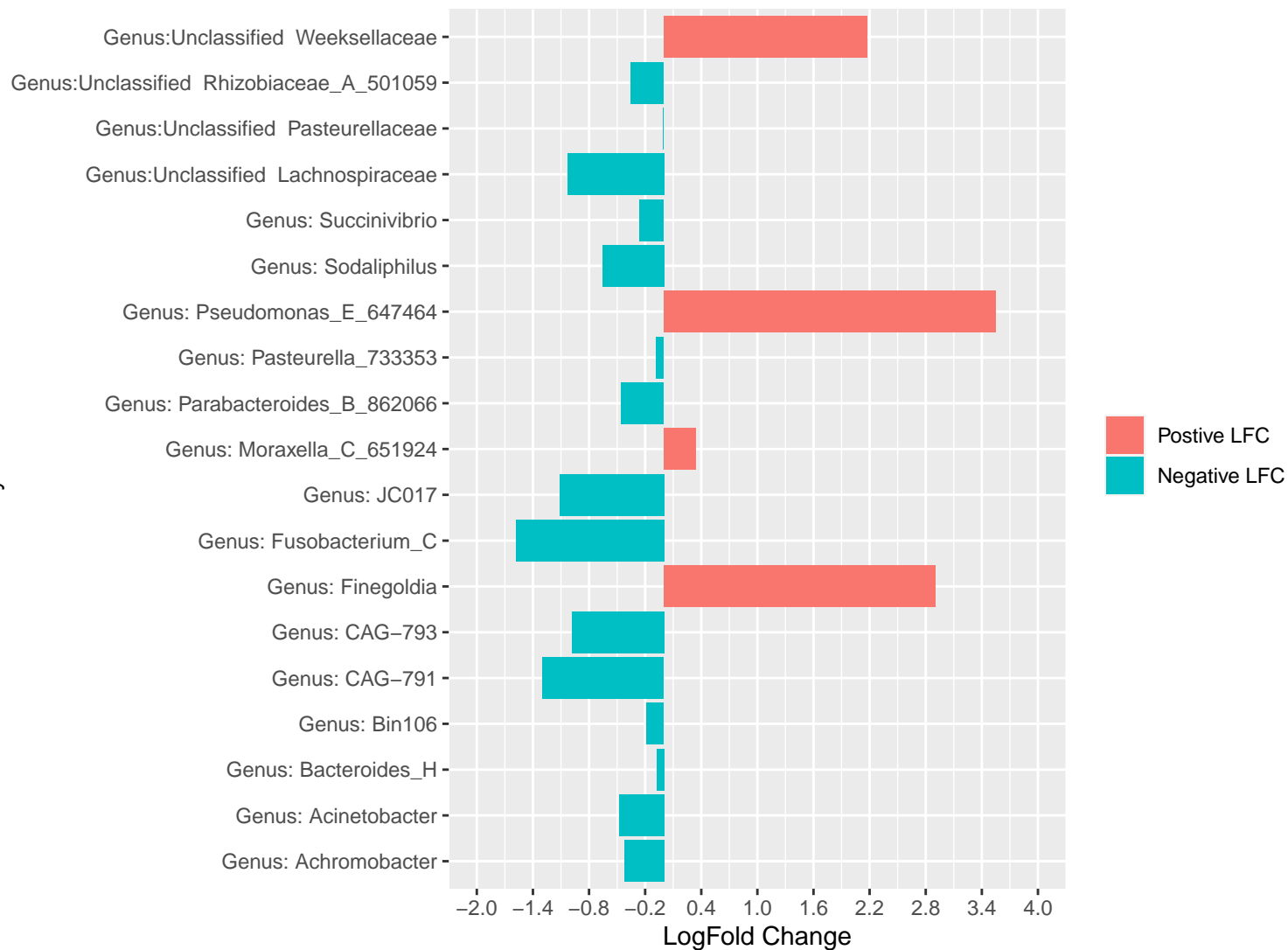

Supplement: Supplementary file 7 [file Data_Sheet_7.pdf]
