# Supplementary material for: Insights into microbial compositions of the respiratory tract of neonatal dairy calves in a longitudinal probiotic trial through 16S rRNA sequencing
Source: Front Microbiol. 2025 Jan 8;15:1499531. doi: 10.3389/fmicb.2024.1499531 (PMC11751226; doi:10.3389/fmicb.2024.1499531)
Supplement: Supplementary file 8 [file Data_Sheet_8.pdf]

```
library("phyloseq")
library("ANCOMBC")
library("tidyverse")
library("gplots")
library("qiime2R")
library("vegan")

##phyloseq workup
#loading feature table
otu <- read.table(file="purdue-feature-table.tsv",
sep="\t",header=T,row.names=1,skip=1,comment.char="", check.names = FALSE)
#loading taxa classifier
tax <- read.table(file="taxonomy.tsv",sep="\t",header=T,row.names=1)
#clean the taxa, Greengenes format
taxa <- tax %>% select(Taxon) %>%
separate(Taxon,c("Kingdon","Phylum","Class","Order","Family","Genus","Species"),";")
taxa.clean <- data.frame(row.names = row.names(taxa),
                         Kingdom = str_replace(taxa[,1], "d__",""),
                         Phylum = str_replace(taxa[,2], "p__",""),
                         Class = str_replace(taxa[,3], "c__",""),
                         Order = str_replace(taxa[,4], "o__",""),
                         Family = str_replace(taxa[,5], "f__",""),
                         Genus = str_replace(taxa[,6], "g__",""),
                         Species = str_replace(taxa[,7], "s__",""),
                         stringsAsFactors = FALSE)
taxa.clean[is.na(taxa.clean)] <- ""
taxa.clean[taxa.clean=="__"] <- ""

for (i in 1:nrow(taxa.clean)){
  if (taxa.clean[i,7] != ""){
    taxa.clean$Species[i] <- paste(taxa.clean$Genus[i], taxa.clean$Species[i], sep = " ")
  } else if (taxa.clean[i,2] == ""){
    kingdom <- paste("Unclassified", taxa.clean[i,1], sep = " ")
    taxa.clean[i, 2:7] <- kingdom
  } else if (taxa.clean[i,3] == ""){
    phylum <- paste("Unclassified", taxa.clean[i,2], sep = " ")
    taxa.clean[i, 3:7] <- phylum
  } else if (taxa.clean[i,4] == ""){
    class <- paste("Unclassified", taxa.clean[i,3], sep = " ")
    taxa.clean[i, 4:7] <- class
  } else if (taxa.clean[i,5] == ""){
    order <- paste("Unclassified", taxa.clean[i,4], sep = " ")
    taxa.clean[i, 5:7] <- order
  } else if (taxa.clean[i,6] == ""){
    family <- paste("Unclassified", taxa.clean[i,5], sep = " ")
    taxa.clean[i, 6:7] <- family
  } else if (taxa.clean[i,7] == ""){
```

```
    taxa.clean$Species[i] <- paste("Unclassified ",taxa.clean$Genus[i], sep = " ")
  }
}

taxa.clean <- taxa.clean[,2] !="Unassigned"
metadata <-read.table(file="purdue_manifest.txt",sep="\t",header =T, row.names
=NULL)
OTU <- otu_table(as.matrix(otu),taxa_are_rows=TRUE)
TAXA <- tax_table(as.matrix(taxa.clean))
SAMPLE <- sample_data(metadata)
TREE <- read_tree("purdue_tree.nwk")

#Generate a phyloseq object
ps_subject <- phyloseq(OTU,TAXA,SAMPLE,TREE)

## phyloseq-class experiment-level object
## otu_table()   OTU Table:         [ 136551 taxa and 301 samples ]
## sample_data() Sample Data:       [ 301 samples by 7 sample variables ]
## tax_table()   Taxonomy Table:    [ 136551 taxa by 7 taxonomic ranks ]
## phy_tree()    Phylogenetic Tree: [ 136551 tips and 128626 internal nodes ]


otu_sums <- colSums(OTU)
average_reads <- mean(otu_sums)
cat("Average number of reads per sample:", average_reads, "\n")
min_reads <- min(otu_sums)
max_reads <- max(otu_sums)
cat("Minimum reads across samples:", min_reads, "\n")
cat("Maximum reads across samples:", max_reads, "\n")
sample_read_counts <- data.frame(Sample = names(otu_sums), Read_Count =
otu_sums)
print(sample_read_counts)

ps_subject1 <- prune_taxa(rowSums(otu_table(ps_subject) == 0) <
ncol(otu_table(ps_subject)) * 0.9, ps_subject)

#Subset based on timepoint and bodysites

ps_T <- subset_samples(ps_subject, Site%in%c("T"))
ps_N <- subset_samples(ps_subject, Site%in%c("N"))

unique_days <- unique(sample_data(ps_T)$Day)

for (day in unique_days) {
  object_name <- paste0("ps_",day,"T")
  subset_ps <- subset_samples(ps_T, Day == day)
  assign(object_name, subset_ps)
```

```
}

ls(pattern = "ps_\\d+T")
#repeat above steps for Nostril

#Check abundance
ps.phylum = tax_glom(ps_subject1, taxrank="Phylum", NArm=FALSE)

plot_bar(ps_subject1, fill="Phylum") +facet_wrap(~Site, scale="free_x", nrow=1)


##for qiime2R visualization and stats##

#Reading datatable
SVs <- read_qza("purdue_gg_table.qza")$data
names(SVs)
SVs$data[1:5,1:5]
SVs$uuid
SVs$type
SVs$contents
SVs$provenance

#Reading metadata
meta <- read.table(file="../purdue_manifest_new1.txt",sep="\t",header =T, row.names
=NULL)
meta <- meta[-c(302:315),]
meta$Sample.ID <- paste("S",meta$Sample.ID, sep="")
head(meta)

#Reading taxonomy
taxonomy<-read_qza("taxa_purdue_gg.qza")$data %>% parse_taxonomy()
taxonomy_copy <- taxonomy[taxonomy$Kingdom !="Unassigned", ]
head(taxonomy)
taxasums<-summarize_taxa(SVs,taxonomy_copy)$Genus

##Alpha diversity shannon##

shannon<-read_qza("purdue_diversity/shannon_vector.qza")

shannon<-shannon$data %>% rownames_to_column("Sample.ID")
gplots::venn(list(meta=meta$`Sample.ID`,shannon=shannon$Sample.ID))
shannon$Sample.ID <- paste("S",shannon$Sample.ID, sep="")
meta <- meta %>%
  mutate("Sample.ID" = as.character("Sample.ID"))
meta_shannon <- meta %>% left_join(shannon)

meta_shannon$Day <- as.factor(meta_shannon$Day)
```

```
#stats for shannon index
model <- lmer(shannon_entropy ~ Site * Group * Day + (1 | Animal), data =
meta_shannon)
summary(model)
emmeans_result <- emmeans(model, pairwise ~ Site * Group | Day)
emmeans_result
emmeans_df <- as.data.frame(summary(emmeans_result$contrasts))
emmeans_df <- emmeans_df[complete.cases(emmeans_df), ]

#To visualize shannon index using line graph
meta_shannon %>% ggplot(aes(x=Day,y=shannon_entropy,color=Site,shape=Group))+
  stat_summary(fun.data = mean_cl_normal, geom = "errorbar", fun.args = list(mult = 1))
+
  stat_summary(geom="line",fun.data = mean_se) +
  stat_summary(geom="point",fun.data = mean_se, size=4) +
  xlab("Days") +
  ylab("Shannon Diversity") +
  theme_q2r() +
  scale_color_viridis_d(name="Body Site")

ggsave("Shannon_by_time_cl.pdf", height=3, width=4, device="pdf")

##bar graph for shannon##
meta %>%
  ggplot(aes(x=Group, y=shannon_entropy, fill=Group)) +
  stat_summary(geom="bar", fun.data=mean_se, color="black") +
  geom_jitter(shape=21, width=0.2, height=0) +
  coord_cartesian(ylim=c(2,7)) +
  facet_grid(~Site+Day) +
  xlab("Probiotic Treatment") +
  ylab("Shannon Diversity") +
  theme_q2r() +
  scale_fill_manual(values=c("slategray3","mistyrose2")) +
  theme(legend.position="none")

ggsave("Shannon_by_Treatment_day.pdf",height=8,width=14,device="pdf")

##Beta diversity##
#PCoA with unweighted unifrac as distance

uwunifrac <- read_qza("purdue_diversity/unweighted_unifrac_pcoa_results.qza")
colnames(uwunifrac$data$Vectors)
colnames(uwunifrac$data$Vectors)[1] <-"Sample.ID"
uwunifrac$data$Vectors$Sample.ID <- paste("S",uwunifrac$data$Vectors$Sample.ID,
sep="")
uwunifrac$data$Vectors
```

```
#with shannon index
uwunifrac$data$Vectors %>%
  select(Sample.ID, PC1, PC2) %>%
  left_join(meta) %>%
  left_join(shannon) %>%
  ggplot(aes(x=PC1, y=PC2, color=Site, shape=Group, size=shannon_entropy)) +
  geom_point(alpha=0.5) +
  theme_q2r() +
  scale_shape_manual(values=c(16,1), name="Probiotic Treatment") +
  scale_size_continuous(name="Shannon Diversity") +
  scale_color_discrete(name="Body Site")
ggsave("PCoA_all_withshannon.pdf", height=4, width=5, device="pdf")

#without shannon index
uwunifrac$data$Vectors %>%
  select(Sample.ID, PC1, PC2) %>%
  left_join(meta) %>%
  ggplot(aes(x=PC1, y=PC2, color=Site, shape=Group)) +
  geom_point(alpha=0.5) +
  theme_q2r() +
  scale_shape_manual(values=c(16,1), name="Probiotic Treatment") +
  #scale_size_continuous(name="Shannon Diversity") +
  scale_color_discrete(name="Body Site")
ggsave("PCoA_bodysite_withoutshannon.pdf", height=4, width=5, device="pdf")

h <- how(plots = Plots(strata = meta$Animal, type = "none"), nperm = 499)
adonis_result <- adonis2(unifrac_dist ~ Site+Group, data = meta, permutations = h)

#PCoA using the weighted unifrac as distance

wunifrac <- read_qza("purdue_diversity/weighted_unifrac_pcoa_results.qza")
colnames(wunifrac$data$Vectors)
colnames(wunifrac$data$Vectors)[1] <-"Sample.ID"
wunifrac$data$Vectors

#with shannon index
wunifrac$data$Vectors %>%
  select(Sample.ID, PC1, PC2) %>%
  left_join(meta) %>%
  left_join(shannon) %>%
  ggplot(aes(x=PC1, y=PC2, color=Site, shape=Group, size=shannon_entropy)) +
  geom_point(alpha=0.5) +
  theme_q2r() +
  scale_shape_manual(values=c(16,1), name="Probiotic Treatment") +
  scale_size_continuous(name="Shannon Diversity") +
  scale_color_discrete(name="Body Site")
```

```
ggsave("PCoA_all_withshannon.pdf", height=4, width=5, device="pdf")

#without shannon index
wunifrac$data$Vectors %>%
  select(Sample.ID, PC1, PC2) %>%
  left_join(meta) %>%
  ggplot(aes(x=PC1, y=PC2, color=Site, shape=Group)) +
  geom_point(alpha=0.5) +
  theme_q2r() +
  scale_shape_manual(values=c(16,1), name="Probiotic Treatment") +
  scale_color_discrete(name="Body Site")
ggsave("PCoA_bodysite_weighted.pdf", height=4, width=5, device="pdf")


##Relative abundance plot##
#heatmap

taxa_heatmap <- taxa_heatmap(taxasums,meta,"Site")
taxa_heatmap <-taxa_heatmap+facet_grid(~Day+Site+Group,scales= "free_x",
space="free_x")
taxa_heatmap

ggsave("heatmap_abudance.pdf", height=12, width=22, device="pdf")


#barplot
meta_subset_tonsil <- meta[meta$Site=="T",]
meta_subset_nostril <- meta[meta$Site=="N",]

taxasums_tonsil_cols <- colnames(taxasums)[colnames(taxasums) %in%
rownames(meta_subset_tonsil)]
taxasums_tonsil <- taxasums[, taxasums_tonsil_cols]
taxasums_nostril_cols <- colnames(taxasums)[colnames(taxasums) %in%
rownames(meta_subset_nostril)]
taxasums_nostril <- taxasums[, taxasums_nostril_cols]

taxa_barplot_nostril <- taxa_barplot(taxasums_nostril,meta_subset_nostril)
taxa_barplot_nostril <-taxa_barplot_nostril_filter1+facet_grid(~Day+Group+Site,scales=
"free_x", space="free_x")
taxa_barplot_nostril
ggsave("abundance_nostril.pdf", height = 12, width=22, device="pdf")

taxa_barplot_tonsil <- taxa_barplot(taxasums_tonsil,meta_subset_tonsil)
taxa_barplot_tonsil <-taxa_barplot_tonsil_filter+facet_grid(~Day+Group+Site,scales=
"free_x", space="free_x")
taxa_barplot_tonsil
ggsave("abundance_tonsil.pdf", height = 12, width=22, device="pdf")
```

```
taxa_barplot <- taxa_barplot(taxasums,meta,"Site")
taxa_barplot <-taxa_barplot+facet_grid(~Day+Group+Site,scales= "free_x",
space="free_x")
taxa_barplot
ggsave("abundance_all.pdf", height = 12, width=22, device="pdf")

#RA table
meta_subsets <- split(meta, meta$Day)
for (day in names(meta_subsets)) {
  assign(paste0("meta_Day_", day), meta_subsets[[day]])
}

unique_days <- unique(meta$Day)
day_summaries <- list()
for (day in unique_days) {
  meta_day <- meta[meta$Day == day, ]
  taxasums_cols <- colnames(taxasums_subset)[colnames(taxasums_subset) %in%
rownames(meta_day)]
  taxasums_subset_day <- taxasums_subset[, taxasums_cols]
  taxasums_day_no_zero <- taxasums_subset_day[rowSums(taxasums_subset_day !=
0) > 0, ]
  taxasums_day_RA <- taxasums_day_no_zero %>% mutate(across(everything(), ~ .x /
sum(.x, na.rm = TRUE) * 100))
  day_avg_RA <- rowMeans(taxasums_day_RA, na.rm = TRUE)
  day_summary <- data.frame(
    Taxon = rownames(taxasums_day_RA),
    Avg_RA = day_avg_RA
  )
  day_summaries[[paste0("Day_", day)]] <- day_summary
}
for (day in names(day_summaries)) {
  write_xlsx(day_summaries[[day]], paste0(day, "_avg_RA.xlsx"))
}

##Differential abundance##
#Ancombc

ps.taxa_0_N <- tax_glom(ps_0_N,taxrank='Genus',NArm=FALSE)

out_0_T = ancombc(data = ps.taxa_0_T, formula = "Group",
                  tax_level = "Genus",
                  p_adj_method = "holm", lib_cut = 1000, prv_cut = 0.1,
                  group = "Group", struc_zero = TRUE, neg_lb = FALSE,
                  tol = 1e-5, max_iter = 100, conserve = FALSE, alpha = 0.05, global =
FALSE)
res_0_T<-out_0_T$res
```

```
#put results in a dataframe
tab_0_T <- res_0_T$q_val
col_name_0_T <- colnames(tab_0_T)
tab_0_T %>% datatable(caption="Adjusted p-values from the Primary Results") %>%
formatRound(col_name_0_T[-1],digits=4)
#Differentially abundant taxa
tab_diff_0_T <- res_0_T$diff_abn
col_name_0_T <- colnames(tab_diff_0_T)
tab_diff_0_T %>% datatable(caption="Differentially Abundant Taxa from the Primary
Results")

ancombc_0_T <- data.frame(
  Species = res_0_T$lfc[,c(1)],
  lfc = unlist(res_0_T$lfc),
  se = unlist(res_0_T$se),
  W = unlist(res_0_T$W),
  p_val = unlist(res_0_T$p_val),
  q_val = unlist(res_0_T$q_val),
  diff_abn = unlist(res_0_T$diff_abn))

ancombc_0_T <- ancombc_0_T %>%
  dplyr::filter(diff_abn == "TRUE")
ancombc_0_T <- ancombc_0_T %>%
  dplyr::filter(grepl("GroupTreatment",rownames(ancombc_0_T)))

#visualization for DA analysis
plt35t <-ggplot(data = ancombc_35_T,aes(y=as.numeric(lfc),x=Species,fill=lfc<0))+
        geom_bar(stat="identity", width=0.6, position=position_dodge(0.9))+
        geom_col()+
        scale_fill_discrete(labels=c("Postive LFC","Negative LFC"))+
        theme(legend.title=element_blank())+
        scale_y_continuous(breaks=seq(-3,2,0.6),limits=c(-3,2))+
        ylab("LogFold Change")+
        xlab("")+
        ggtitle(c('Day 35'))+
        coord_flip()
plt35t
#repeat for all results
#merge
plt0t <-plt0t+labs(tag="A")+theme(plot.tag=element_text())
plt7t <-plt7t+labs(tag="B")+theme(plot.tag=element_text())
plt14t <-plt14t+labs(tag="C")+theme(plot.tag=element_text())
plt21t <-plt21t+labs(tag="D")+theme(plot.tag=element_text())
plt28t <-plt28t+labs(tag="E")+theme(plot.tag=element_text())
plt35t <-plt35t+labs(tag="F")+theme(plot.tag=element_text())
plt42t <-plt42t+labs(tag="G")+theme(plot.tag=element_text())
```

```
plt49t <-plt49t+labs(tag="H")+theme(plot.tag=element_text())
plt_t_all<-grid.arrange(plt0t,plt7t,plt14t,plt21t,plt28t,plt35t,plt42t,plt49t, left="Differentially
Abundant Genus", top="Tonsil Treated vs Control Samples")
```
